# Supplementary material for: Spatiotemporal characteristics and optimization strategies of land use and land resource carrying capacity in the three gorges reservoir region (1986–2020)
Source: PLoS One. 2025 Aug 21;20(8):e0330461. doi: 10.1371/journal.pone.0330461 (PMC12370055; doi:10.1371/journal.pone.0330461)
Supplement: Attachment — Submitted filename: Response to Reviewers.docx [file pone.0330461.s002.docx]

Point to Point Responses

**Paper Ref. No.:PONE-D-24-37148**

**Title:**Research on the Spatiotemporal Characteristics and Optimization Strategies of Land Use and Land Resource Carrying Capacity in the Three Gorges Reservoir Region over the Past 30 Years.

Thanks so much for all the kind help to this manuscript. We have analyzed the valuable comments from the editor and two independent reviewers carefully, and tried our best to revise the manuscript. We appreciate the thoughtful review and constructive feedback provided by tow viewers.We agree with the reviewer's suggestions and have incorporated the recommended changes into the manuscript.These comments have improved the quality of the paper immensely. The point to point responses are listed as follows.

# Editor

**1.Comment:**

Please ensure that your manuscript meets PLOS ONE's style requirements, including those for file naming.The PLOS ONE style templates can be found at

https://journals.plos.org/plosone/s/file?id=wjVg/PLOSOne_formatting_sample_main_body.pdf and https://journals.plos.org/plosone/s/file?id=ba62/PLOSOne_formatting_sample_title_authors_affiliations.pdf

**Revisions：**

Thank you very much for reviewing our manuscript and providing valuable feedback. We have carefully reviewed the formatting requirements of PLOS ONE and compared them with the templates provided in the links you shared. Herein, we confirm that we have made corresponding adjustments to the manuscript to ensure it fully complies with the style and formatting standards of PLOS ONE, including all details such as file naming.

**2.Comment:**

We note that the grant information you provided in the‘Funding Information’and‘Financial Disclosure’sections do not match. When you resubmit, please ensure that you provide the correct grant numbers for the awards you received for your study in the‘Funding Information’section.

**Revisions：**

Thank you very much for reviewing our manuscript and pointing out the inconsistency in the funding information between the "Funding Information" and "Financial Disclosure" sections. We have carefully checked and corrected the grant numbers to ensure that both sections are now consistent and accurately reflect the funding we received for our research. Once again, thank you for your patient guidance.The specific modifications are outlined below:

“***Funding*** *This research work was partly supported by the National Social Science Funds of China under Grants No.22XJY006,and the Social Science Research Project of Chongqing Municipal Education Commission under Grants No.24SKGH361.”*

**3.Comment:**

Thank you for stating in your Funding Statement: "This research work was partly supported by the National Social Science Funds of China under Grants No.22XJY006,and Science and Technology Research Program of Chongqing Municipal Education Commission under Grants No.KJQN202002101."Please provide an amended statement that declares *all* the funding or sources of support (whether external or internal to your organization) received during this study, as detailed online in our guide for authors at http://journals.plos.org/plosone/s/submit-now. Please also include the statement “There was no additional external funding received for this study.” in your updated Funding Statement. Please include your amended Funding Statement within your cover letter. We will change the online submission form on your behalf.

**Revisions：**

Thank you for pointing out the need for improvement in the funding statement. I have revised the funding statement according to the PLOS ONE guidelines (http://journals.plos.org/plosone/s/submit-now). I confirm that this research was supported by the National Social Science Funds of China (Grant No. 22XJY006) and the Social Science Research Project of Chongqing Municipal Education Commission (Grant No. 24SKGH361). I also declare that no additional external funding was received for this study. The revised statement has been included in Submission Cover Letter 2. Please find it there,thank you once again.The specific modifications are outlined below:

“*This study was supported by the National Social Science Funds of China under Grants No.22XJY006,and the Social Science Research Project of Chongqing Municipal Education Commission under Grants No.24SKGH361, which only provided support for data collection in this study.The funders did not play a role in research design, data analysis, publication decisions, or manuscript preparation.”*

**4.Comment:**

Thank you for stating the following financial disclosure: "This research work was partly supported by the National Social Science Funds of China under Grants No.22XJY006,and Science and Technology Research Program of Chongqing Municipal Education Commission under Grants No.KJQN202002101."Please state what role the funders took in the study. If the funders had no role, please state: ""The funders had no role in study design, data collection and analysis, decision to publish, or preparation of the manuscript."" If this statement is not correct you must amend it as needed. Please include this amended Role of Funder statement in your cover letter; we will change the online submission form on your behalf.

**Revisions：**

Thank you sincerely for your invaluable comments and suggestions. According to the suggestion, We explained in the submission letter the role played by the funding fund. The specific content is: 'This study was supported by the the National Social Science Funds of China (Grant No. 22XJY006) and the Social Science Research Project of Chongqing Municipal Education Commission (Grant No. 24SKGH361), which only provided support for data collection in this study.The funders did not play a role in research design, data analysis, publication decisions, or manuscript preparation.

**5.Comment:**

Please include a separate caption for each figure in your manuscript.

**Revisions：**

Thank you very much for your review comments. We have added independent and clear captions to each figure in the manuscript as required, describing the content of the figures, their relevance to the article's theme, and their role in the argumentation. The revised manuscript has been re-uploaded for your review.

**6.Comment:**

Please review your reference list to ensure that it is complete and correct. If you have cited papers that have been retracted, please include the rationale for doing so in the manuscript text, or remove these references and replace them with relevant current references. Any changes to the reference list should be mentioned in the rebuttal letter that accompanies your revised manuscript. If you need to cite a retracted article, indicate the article’s retracted status in the References list and also include a citation and full reference for the retraction notice.

**Revisions：**

Thank you very much for your review comments. We have thoroughly examined the reference list to ensure its completeness and correctness. For any citations of retracted papers, we have addressed them accordingly, either by explaining the specific rationale for the citation within the manuscript or by replacing them with relevant current references. All changes have been highlighted in the "Revised Manuscript with Track Changes," and the revised manuscript has been attached for your review.

**7.Comment:**

1. We note that Figures 1, 2 and 3 in your submission contain [map/satellite] images which may be copyrighted. All PLOS content is published under the Creative Commons Attribution License (CC BY 4.0), which means that the manuscript, images, and Supporting Information files will be freely available online, and any third party is permitted to access, download, copy, distribute, and use these materials in any way, even commercially, with proper attribution. For these reasons, we cannot publish previously copyrighted maps or satellite images created using proprietary data, such as Google software (Google Maps, Street View, and Earth). For more information, see our copyright guidelines: <http://journals.plos.org/plosone/s/licenses-and-copyright.>

**Revisions：**

Thank you very much for pointing out the copyright issue of the maps in Figures 1, 2, and 3. We have immediately added the sources of the maps after the figure titles. Specifically, these data are sourced from publicly and freely available resources, involving no copyright disputes, to ensure that the use of all images complies with PLOS's copyright regulations. Thank you again for your thorough review and professional guidance.The specific modifications are outlined below:

***Fig. 1 Research framework diagram.****Note:The basemap was obtained from the Geospatial Data Cloud(<http://www.gscloud.cn/home>), and the map boundary has not been changed.Cartographic software:ArcGIS 10.8.*

***Fig. 2 Location Map of the TGRR****.Note:DEM data is sourced from the Geospatial Data Cloud website (http://www.gscloud.cn/home).Administrative boundary data comes from Resource and Environment Science and Data Center (https://www.resdc.cn/),and the map boundary has not been changed.Cartographic software:ARCGIS 10.8.*

***Fig. 3 Land use status map of the TGRR from 1986 to 2020.****Note:The basemap was obtained from the Geospatial Data Cloud(<http://www.gscloud.cn/home>), and the map boundary has not been changed.Cartographic software:ArcGIS 10.8.*

# Reviewer #1

Thanks very much for your comments. According to the following valuable comments and suggestions, we have tried our best to revise the manuscript, and these comments have substantially improved the quality of the paper.

**1.Comment:**

Abstract Improvements

Page 1, Line 12-20:

Comment: The abstract could benefit from more detail regarding the classification maps and specific methodology used for land use changes and evaluation. Consider adding a brief mention of the tools used for remote sensing (e.g., ARCGIS version, Landsat processing steps).

Suggestion: Add the following: “Classified maps were generated using ARCGIS 10.8, and Landsat TM images were processed for accuracy using supervised classification techniques.”

**Response:**

Thank you for your suggestion.We have noticed that the abstract lacked sufficient details in describing the classified maps and the specific methodologies used for land use change and evaluation.In accordance with your advice, we have added relevant information,clearly stating that the classified maps were generated using ARCGIS 10.8, and that Landsat TM images were processed for accuracy using supervised classification techniques, in order to enhance the completeness and clarity of the abstract. The specific modifications are outlined below:

*“Abstract*

*Studying land use changes caused by human economic activities is beneficial for the sustainable growth, making it a global research hotspot. In this study, we used Landsat Thematic Mapper images and statistical yearbooks from 1986, 1995, 2000, 2007, 2010, and 2020 to obtain vector and socioeconomic information on the land use status of the Three Gorges Reservoir Region (TGRR) in the corresponding years. We introduced research models on land use quantitative changes, dynamic indicators, and degree index to quantitatively investigate spatiotemporal variations in land use in the TGRR over the past 30 years.Classified maps were generated using ARCGIS 10.8, and Landsat TM images were processed for accuracy using supervised classification techniques.* *Based on the status quo of this region and the analytic hierarchy process, we constructed a land resource carrying ability evaluation indicator model in terms of social, economic, population, and ecological carrying abilities.”*

**2.Comment:**

Methodology Clarity
Page 6, Line 85-92:
Comment: The methodology for Landsat Thematic Mapper image processing lacks details about the specific classification algorithms (e.g., supervised/unsupervised), accuracy assessment, and validation methods. It is crucial to discuss how you ensured the quality of classification.
Suggestion: Include details on the classification methods and any accuracy metrics (e.g., overall accuracy, Kappa statistics). A sentence such as "The land use types were classified using a supervised classification method, and the overall accuracy of the classification was validated with ground truth data, achieving an accuracy of XX%."

**Response:**

Thank you very much for the valuable comments from the reviewer. In response to your point about the lack of details in the methodology for Landsat Thematic Mapper image processing regarding specific classification algorithms, accuracy assessment, and validation methods, we have added the following information: "The land use types were classified using a supervised classification method, and the accuracy comparison results based on 688 randomly selected ground truth sample points showed an accuracy of 95.82%. The Kappa coefficient was also highly consistent, thus confirming the accuracy and scientific value of the data." The specific modifications are outlined below:

*“3.2. Data Sources*

*The basic In this study, data of Landsat Thematic Mapper false-color remote sensing images and statistical yearbooks (social, economic, population, and ecological) for six years (1986, 1995, 2000, 2007, 2010, and 2020) were used.The land use types were classified using a supervised classification method, and the accuracy comparison results based on 688 randomly selected ground truth sample points showed an accuracy of 95.82%. The Kappa coefficient was also highly consistent, thus confirming the accuracy and scientific value of the data.”*

**3.Comment:**

Classified Map and Figures

Page 9, Line 173-175:

Comment: The classified maps in Figure 3 and Table 3 need to be referenced more explicitly in the text. Ensure the map resolution is high enough for clear interpretation of land use changes.

Suggestion: Add a sentence in the results section like, "Figure 3 shows the classified maps of land use change over the years, clearly indicating the spatial transformation of the landscape in the TGRR." Additionally, consider improving the map legends to make the classification clearer.

**Response:**

Thank you very much for your review comments. We deeply appreciate your suggestions on enhancing the clarity and interpretability of the classified maps in Figure 3 and Table 3. To address your concerns, we have already incorporated a more explicit reference in the results section, stating: "Figure 3 shows the classified maps of land use change over the years, clearly illustrating the spatial transformation of the landscape in the Three Gorges Reservoir Region (TGRR)." Additionally, we recognize the significance of map resolution for clear interpretation and will ensure that the maps are of high quality, allowing for detailed examination of land use changes. To further improve clarity, we have revised the map legends to enhance their readability. The specific modifications are outlined below:

*“3.1. Quantitative Changes of Land Use Types*

*Spatial information technology was used to statistically analyze land use status in the TGRR from 1986 to 2020. The results are presented in Table 3. Using the mapping function of ARCGIS 10.8 software, a classification map of the land use status was created.Figure 3 shows the classified maps of land use change over the years, clearly indicating the spatial transformation of the landscape in the TGRR.Table 3 presents the area and proportion of different land use types over the years, providing a clear view of the changes in the quantities of various land uses within the study area.”*

This is the revised Fig 3, with the font size of the legend increased from the previous 36 points to the current 48 points for better clarity.

Fig.3

**4.Comment:**

Data Availability and Completeness

Page 6, Line 87:

Comment: The source of socioeconomic data is not clearly described. While it mentions "statistical yearbooks," it is important to explain whether data were consistently available across all years (1986-2020). Gaps in the data, if any, should be acknowledged.

Suggestion: Include a note such as, "Socioeconomic data from certain years had limited availability and were interpolated using trend analysis for consistency."

**Response:**

Thank you for your valuable suggestion. In response to your recommendation, we have made corresponding revisions to the paper. In the "3.2. Data Sources" section, we have clearly stated: "Socioeconomic data from certain years had limited availability, and to maintain consistency, we used trend analysis for interpolation." This explanation aims to ensure that readers understand how we addressed the issue of missing data and how we ensured the completeness and availability of the data.The specific modifications are outlined below:

*“3.2. Data Sources*

*The basic In this study, data of Landsat Thematic Mapper false-color remote sensing images and statistical yearbooks (social, economic, population, and ecological) for six years (1986, 1995, 2000, 2007, 2010, and 2020) were used.In addition.Socioeconomic data from certain years had limited availability and were interpolated using trend analysis for consistency.The land use types were classified using a supervised classification method, and the accuracy comparison results based on 688 randomly selected ground truth sample points showed an accuracy of 94.52%. The Kappa coefficient was also highly consistent, thus confirming the accuracy and scientific value of the data.”*

**5.Comment:**

Accuracy Assessment Missing

Page 11, Line 195-200:

Comment: The accuracy assessment of the classified land use map and model performance is missing. Readers need to understand how reliable the land classification is over time.

Suggestion: Include a section discussing how you validated the classification, such as "Accuracy assessment of classified maps was conducted using confusion matrices and cross-validation, achieving an overall accuracy of XX% for land use types."

**Response:**

Thank you for your valuable suggestion. We fully understand the reviewer's concern regarding the absence of an accuracy assessment section and acknowledge its crucial role in evaluating the reliability of land use classification. After careful consideration of your comments, we have made corresponding supplements and improvements to our study (Section 3.3. Accuracy Assessment of Land Use Classification Maps and Model Performance). Specifically, we have added a section on the accuracy assessment of the classification, detailing how the classification results were validated. We employed the confusion matrix method to evaluate the accuracy of the classified maps and calculated the overall accuracy for different land use types. These results have now been incorporated into the revised paper. With this addition, we aim to provide readers with more comprehensive information, enabling them to understand the reliability of our land use classification at different time points. We sincerely appreciate your suggestions, which have significantly enhanced the completeness and credibility of our research. An overview of the specific modifications is provided below:.The specific modifications are outlined below:

*“3.3. Accuracy Assessment of Land Use Classification Maps and Model Performance*

*In this study, we place great emphasis on the accuracy assessment of land use classification, as it is a key indicator of the reliability and effectiveness of our classification method. To ensure that readers can fully understand the reliability of land classification over time, we conducted a comprehensive accuracy assessment of the classification results using the confusion matrix validation method. Specifically, we used the confusion matrix to calculate the user accuracy, producer accuracy, overall accuracy, and Kappa Coefficient for five time intervals (1986-2020) in the study area. These indicators can intuitively reflect the consistency between the classification results and actual observed data. By comparing the classification results over different time periods, we found that although there were some fluctuations, the overall accuracy remained at a high level. For instance, in the five classification periods, we achieved an average overall accuracy of 92.14% and an average Kappa Coefficient of 88.47%, indicating that our classification method has high stability and reliability. These evaluation results not only prove that our classification method is accurate and reliable but also provide readers with an important basis for understanding the reliability of land classification changes over time. Detailed data are presented in Table 5.”*

***Table 5*** *Accuracy Validation Statistics Table for Land Use Classification Confusion Matrix.*

| ***Year/Accuracy*** | ***User Accuracy（Arithmetic Mean）*** | ***Producer Accuracy***  ***(Arithmetic Mean)*** | ***Overall Accuracy*** | ***Kappa Coefficient*** |
| --- | --- | --- | --- | --- |
| *1986-1995* | *91.12%* | *90.28%* | *91.64%* | *88.55%* |
| *1995-2000* | *88.69%* | *89.27%* | *91.51%* | *87.26%* |
| *2000-2007* | *90.83%* | *91.61%* | *93.13%* | *89.14%* |
| *2007-2010* | *87.95%* | *88.62%* | *92.59%* | *89.47%* |
| *2010-2020* | *89.49%* | *90.83%* | *91.84%* | *87.93%* |

**6.Comment:**

Land Use Dynamics

Page 12, Line 203-210:

Comment: While the dynamic index model is presented, it is not clear how the transitions between different land types were handled in the spatial-temporal analysis. You should elaborate on how changes between forest, arable, and construction land were treated in the model.

Suggestion: Add details like, "The transition between land types was modeled using temporal change detection algorithms in ARCGIS, and transitions were cross-referenced with historical land use maps."

**Response:**

Thank you for your valuable comment. In response, we have added relevant details in the section "3.2. Speed of Land Use Variation in the TGRR," clarifying that the transition between land types was modeled using temporal change detection algorithms in ARCGIS, and these transitions were cross-referenced with historical land use maps to ensure accuracy in our spatial-temporal analysis.The specific modifications are outlined below:

*“3.2. Speed of Land Use Variation in the TGRR*

*Using Equations 1 and 2, the rate of land use variation in the TGRR was analyzed, and the single and comprehensive dynamic levels of every region over the past 30 years were obtained, as shown in Table 4.In this study, the approach for handling transitions between different types of land involved modeling the conversion between land types using temporal change detection algorithms in ARCGIS, and cross-referencing these transitions with historical land use maps for verification. ”*

**7.Comment:**

Evaluation and Model Explanation

Page 7, Line 124-130:

Comment: The construction of the evaluation index system (AHP and mean-square decision-making approach) is not explained clearly. It is important to detail the rationale behind choosing certain indicators and their respective weights.

Suggestion: Expand this section with the following: "The selection of indicators for the land resource carrying capacity was based on an analytic hierarchy process (AHP), with weights determined using a mean-square decision-making approach. Each criterion was verified by experts in the field for robustness."

**Response:**

Thank you very much for your valuable suggestions. In response to your feedback, we have supplemented and explained relevant content in the "3.3.3. Land Use Index Model" section, elaborating on the basis for indicator selection. Specifically, the indicators for land resource carrying capacity were selected based on the Analytic Hierarchy Process (AHP), and the weights were determined through the mean-square decision-making method. Each criterion was validated by experts in the field to ensure its robustness.The specific modifications are outlined below:

*“where Ia and I_b_ regional land use indices represent the initial and termination periods, respectively, Ai represents the classification index of the extent of i-level land use, and Cib and Cia represent the extent of i-level land use during the initial and terminal study periods, respectively. This formula can effectively calculate the degree of land use change in the study area.The selection of indicators for the land resource carrying capacity was based on an analytic hierarchy process (AHP), with weights determined using a mean-square decision-making approach. Each criterion was verified by experts in the field for robustness. ”*

**8.Comment:**

Data Gaps and Temporal Resolution

Page 13, Line 259:

Comment: Acknowledge any data gaps during the 30-year period, especially if there are years where Landsat imagery or socioeconomic data might have lower temporal resolution or availability.

Suggestion: Add a statement like, "Due to the limitations in satellite data availability, certain periods required interpolation of land use data, which may introduce minor uncertainties in the analysis."

**Response:**

Thank you very much for the reviewer's valuable comments regarding the data gaps and temporal resolution. In response, we have added a relevant note in the section "3.3.1. Quantitative Changes in Land Use" of our article: "It's worth noting that, due to the limitations in satellite data availability, certain periods required interpolation of land use data, which may introduce some minor uncertainties in the analysis." This statement acknowledges the limitations of the data while ensuring the completeness and accuracy of our analysis.The specific modifications are outlined below:

*“3.3.1. Quantitative Changes in Land Use*

*Studies of the characteristics of land use changes have focused on spatiotemporal variations in land use, including variations in the quantity, spatial distribution, and degree of land use (Teja et al., 2022; Huang et al., 2022). To understand the laws of land use variations, land use status data of the TGRR in 1986, 1995, 2000, 2007, 2010, and 2020 were classified and statistically analyzed spatiotemporally using RS and GIS.It's worth noting that, due to limitations in the availability of satellite data, interpolation of land use data was required for certain periods, which may introduce some minor uncertainties in the analysis.”*

**9.Comment:**

Figures and Tables

Page 14, Line 276:

Comment: Some figures (Figures 4-7) and tables (e.g., Table 6, Table 7) should be more readable, with clearer labels and higher resolution to represent the data accurately. It would be helpful to include error bars or confidence intervals for some of the metrics.

Suggestion: "Figures should include error margins or confidence intervals where applicable to show the variability and reliability of the results."

**Response:**

Thank you very much for taking the valuable time to provide us with detailed and constructive feedback. In response to the concerns raised by the reviewer regarding the resolution and clarity of the figures, we gave it immediate and high attention. To ensure that the figures presented the best possible visual effect, we re-exported all relevant graphics using higher-quality output settings. This improvement not only enhanced the clarity of the figures but also made the details more distinct, greatly improving the readability and comprehensibility of the information, and enabling readers to interpret the key information in the figures more easily.In addition to visually optimizing the figures, we also conducted a thorough and meticulous review of the data in the paper. We fully understand the importance of data accuracy and completeness for research, so we reviewed all the data and ensured their correct citation in the paper. On this basis, we further accurately reflected the confidence intervals of the data in the figures, providing important information about data reliability and uncertainty. This measure not only strengthened the scientificity and rigor of the paper but also provided readers with a more comprehensive perspective for interpreting the data.We are well aware that only through continuous improvement and enhancement can we approach perfection. Therefore, we will continue to strive, constantly exploring and refining various aspects of the paper, in order to present readers with an even more excellent research outcome.The specific modifications are outlined below:

**Fig. 4.** Analysis diagram of population carrying system.

**Fig. 5.** Analysis diagram of economic carrying system.

**Fig. 6.** Analysis diagram of social carrying system.

**Fig. 7.** Analysis diagram of Ecological carrying system.

**10.Comment:**

Recommendations for Future Work

Page 15, Line 360:

Comment: While the recommendations provide a solid direction, the need for higher-resolution satellite imagery (e.g., Sentinel-2) for future studies could be mentioned. This would allow for more precise land use classification.

Suggestion: Add, "Future studies should consider the use of higher-resolution datasets such as Sentinel-2 imagery to enhance the accuracy of land classification and further refine land use change detection."

**Response:**

Thank you very much for your invaluable suggestions. After careful consideration and discussion, we have incorporated the following content into the first key point of the discussion section of our paper, based on the reviewer's insightful feedback: Upon reviewing the recommendations put forward in this study, we recognize that there is still room for improvement in the selection of data sources for future research. Specifically, we recommend the adoption of high-resolution satellite imagery such as Sentinel-2, which offers multispectral band data capable of capturing more subtle features of the earth's surface, thereby exhibiting higher accuracy in land use classification and change detection. This is intended to further enhance the precision and granularity of our research.The specific modifications are outlined below:

*“5. Discussion*

*Based on the panel data, such as remote sensing interpretation data and statistical yearbooks, and with the help of spatial information technologies, such as RS and GIS, and the introduction of research models, such as quantitative changes in the land use, land use dynamic indicators, and land use degree indicators, this study quantitatively analyzed the spatiotemporal variation features of land use in the TGRR over the past 30 years. Based on the status quo of the research area and using the AHP evaluation method, a land resource carrying ability evaluation indicator model was constructed from the dimensions of social, economic, population, and ecological carrying abilities. A mean square error decision-making approach was introduced to determine the weight of the indicators, and the land resource carrying capacity of the TGRR from 1986 to 2020 was comprehensively analyzed. Overall, the research outcomes of this study were objective and credible.*

*(1) The methods used in this study may have certain limitations, mainly because of the subjectivity in the assessment of land resource carrying ability. For example, there may be differences in the research methods, perspectives, and evaluation indicators within the same research areas, resulting in different evaluation results. Nevertheless, the variation patterns of land resource carrying ability in the same region in the same year will be similar and will not affect the comparison of the research results.Upon reviewing the recommendations proposed in this study, we recognize that there is room for further improvement in the selection of data sources for future research. Specifically, while the current study has already provided a clear direction and suggestions for land resource management and sustainable development, in order to enhance the accuracy of land use classification and refine land use change detection, we believe that future research should consider adopting datasets with higher resolution. In this regard, satellite imagery such as Sentinel-2, with its high resolution and wide applicability, has become a highly potential data source. The multispectral band data provided by Sentinel-2 satellites can capture more subtle features of the earth's surface, thereby demonstrating higher accuracy in land use classification and change detection.”*

# We sincerely appreciate the time andeffort invested bythere viewersinevaluatingour manuscript.Welookforward to any additional feedback orsuggestions.

# Reviewer #2

Thanks very much for your comments. According to the following valuable comments and suggestions, we have tried our best to revise the manuscript, and these comments have substantially improved the quality of the paper.

**1.Comment:**

16 indicators were used for evaluation of land carrying capacity, On which basis these indicators were chosen.

**Response:**

Thank you very much for your valuable comments. In response to your suggestions, we have conducted serious thinking and thorough discussions. Overall, the selection of the 16 evaluation factors, including per-capita construction land, per-capita cultivated land area, etc., for this study was based on a combination of previous research findings in the fields of land resource carrying capacity, land use planning, and sustainable development strategies, while also taking full consideration of data availability and practicality to ensure the accuracy and reliability of the evaluation results. Additionally, we took into account the specific conditions of the study area (an important ecological barrier in the upper reaches of a river basin, with dual characteristics of being both a large mountainous region and a large reservoir area). Specifically:Firstly, the selection of these indicators was largely inspired by classic literature in the field of land resource carrying capacity research. For example, Zhang Jinjie's "Research on Rational Utilization and Comprehensive Carrying Capacity of Resources in Fushun City" provides an in-depth discussion of land resource population carrying capacity, land resource construction carrying capacity, land resource economic carrying capacity, and land resource ecological carrying capacity. This has provided us with important guidance for selecting ecological and economic indicators such as per-capita construction land, per-capita cultivated land area, per-capita residential area, forest coverage rate, and per-capita public green space area.Secondly, in the process of evaluating land carrying capacity, we drew on the general framework for land carrying capacity evaluation and analysis. This framework emphasizes the comprehensive consideration of both natural land carrying capacity and human-induced carrying capacity, including natural factors such as land fertility, land type, and topography, as well as human-induced factors such as population density, economic development level, and environmental quality. These factors are highly consistent with the indicators we selected, such as population density, per-capita GDP, per-capita total retail sales of social consumer goods, and the contribution rate of the tertiary industry to GDP.Furthermore, we also referred to domestic and international research findings on land use planning, sustainable development strategies, and urban and environmental management. For example, in the process of urbanization, indicators such as per-capita public service facility land area and urbanization rate are key parameters for measuring the level of urban development and land use efficiency. These indicators have been mentioned in multiple pieces of literature on urban land carrying capacity evaluation.

**2.Comment:**

Please explain what optimization strategies (OS) are used in the research?

**Response:**

Thank you very much for your invaluable feedback, which is of great significance to us in improving our research. Regarding the issue that optimization strategies (OS) were not explicitly mentioned in the study, we have conducted serious reflections and in-depth discussions. Indeed, this was an oversight on our part during the writing process, and we sincerely apologize for it. We acknowledge that this may have had a certain impact on readers' understanding of the comprehensiveness and practicality of the research.After carefully reviewing your review comments, we deeply recognize the importance of optimization strategies in enhancing land resource carrying capacity and promoting regional sustainable development. Therefore, we have decided to add relevant content to the paper to address this shortcoming. Specifically, we have included a new section, Section 8, in Chapter 4, titled "Discussion on Optimization Strategies for Land Carrying Capacity in the Study Area." In this section, we elaborate on the current situation and issues related to land carrying capacity in the study area, and propose optimization strategies and recommendations. These strategies include, but are not limited to: strengthening land use planning and management to improve land use efficiency; intensifying ecological environmental protection efforts to promote ecological restoration and recovery; optimizing industrial structure to drive economic transformation and upgrading; and enhancing population regulation and management to achieve coordinated development between population and resource environment.We believe that by adding this section, we can not only enrich and improve our research but also provide readers with more practical and actionable suggestions, thereby promoting sustainable land use and regional sustainable development in the study area. Once again, thank you for your valuable feedback and corrections. We will continue to strive to improve the quality and standard of our research, and look forward to receiving more guidance and support from you in future studies.The specific modifications are outlined below:

*“4.8. Discussion on Optimization Strategies for Land Carrying Capacity in the Study Area*

*Drawing upon the extensive research and analytical findings of our study, we conclude that optimizing the land carrying capacity of the TGRR and its Upstream Areas (TGRR) necessitates a holistic and multifaceted approach. This entails reinforcing land use planning and management frameworks to guarantee sustainable land utilization and striking a balance among the diverse needs of stakeholders, while embedding the fundamental principles of sustainability, ecological conservation, economic development, and now, green development.Our research highlights the paramount importance of intensifying ecological protection and restoration efforts through initiatives such as afforestation, wetland rehabilitation, and biodiversity preservation, which are vital for sustaining and enhancing the region's ecological resilience. Additionally, promoting green development strategies, such as the adoption of renewable energy sources, energy efficiency improvements, and the integration of green infrastructure into urban planning, will further strengthen the region's commitment to environmental stewardship.Moreover, by transforming the industrial structure towards knowledge-intensive and eco-friendly industries, fostering innovation, and advancing circular economy practices, we can bolster economic competitiveness without compromising environmental integrity. Integrating green technologies and sustainable practices across all economic sectors will ensure that growth is both environmentally sustainable and economically viable.Furthermore, our analysis underscores the necessity of robust population regulation and management strategies, encompassing the implementation of population control policies, the promotion of urbanization paired with rural revitalization, and the enhancement of public services. These measures, coupled with the promotion of green lifestyles and environmental education, are crucial for paving the way towards sustainable development.By meticulously balancing population growth, land use planning, economic development, and green development strategies, our study demonstrates that the TGRR can embark on a trajectory of harmonious and enduring progress. The strategies proposed herein, grounded in rigorous research and comprehensive analysis, are poised to make substantial contributions to the long-term sustainable development of the TGRR and its Upstream Areas, ensuring a future that is both prosperous and environmentally sustainable.”*

**3.Comment:**

Whats the need of OS in spatiotemporal characterization of Land Use?

**Response:**

Thank you very much for your invaluable feedback, which is of great significance to us in improving our research. In the spatiotemporal characterization of land use, the necessity of optimization strategies lies primarily in their ability to more effectively manage and plan land resources, by comprehensively considering both the temporal dimension and spatial layout, thereby enhancing the allocation efficiency of land resources, promoting the protection and sustainable development of the ecological environment, and meeting the needs of economic and social development simultaneously. We will further delve into and refine the relevant strategies to more accurately depict the spatiotemporal characteristics of land use. Detailed descriptions of specific land use strategies are provided in Subsection 8 of Part 4 of our article. Once again, we appreciate your feedback.The specific modifications are outlined below:

*“4.8. Discussion on Optimization Strategies for Land Carrying Capacity in the Study Area*

*Drawing upon the extensive research and analytical findings of our study, we conclude that optimizing the land carrying capacity of the TGRR and its Upstream Areas (TGRR) necessitates a holistic and multifaceted approach. This entails reinforcing land use planning and management frameworks to guarantee sustainable land utilization and striking a balance among the diverse needs of stakeholders, while embedding the fundamental principles of sustainability, ecological conservation, economic development, and now, green development.Our research highlights the paramount importance of intensifying ecological protection and restoration efforts through initiatives such as afforestation, wetland rehabilitation, and biodiversity preservation, which are vital for sustaining and enhancing the region's ecological resilience. Additionally, promoting green development strategies, such as the adoption of renewable energy sources, energy efficiency improvements, and the integration of green infrastructure into urban planning, will further strengthen the region's commitment to environmental stewardship.Moreover, by transforming the industrial structure towards knowledge-intensive and eco-friendly industries, fostering innovation, and advancing circular economy practices, we can bolster economic competitiveness without compromising environmental integrity. Integrating green technologies and sustainable practices across all economic sectors will ensure that growth is both environmentally sustainable and economically viable.Furthermore, our analysis underscores the necessity of robust population regulation and management strategies, encompassing the implementation of population control policies, the promotion of urbanization paired with rural revitalization, and the enhancement of public services. These measures, coupled with the promotion of green lifestyles and environmental education, are crucial for paving the way towards sustainable development.By meticulously balancing population growth, land use planning, economic development, and green development strategies, our study demonstrates that the TGRR can embark on a trajectory of harmonious and enduring progress. The strategies proposed herein, grounded in rigorous research and comprehensive analysis, are poised to make substantial contributions to the long-term sustainable development of the TGRR and its Upstream Areas, ensuring a future that is both prosperous and environmentally sustainable.”*

**4.Comment:**

Similar research studies conducted on the topic have not been cited.

**Response:**

We are deeply grateful for your review and the valuable suggestions you have provided, which are of great significance for us to improve our research. In response to your concern about the insufficient citation of similar studies, we conducted an in-depth re-evaluation and review. During this process, we identified more literature that is closely related to the theme of our study and holds significant value. We have incorporated these references into our bibliography and cited them in the text accordingly. We firmly believe that these newly added citations will further enhance the comprehensiveness and accuracy of our research, while also providing better support and validation for our conclusions. The specific modifications are outlined below:

*“References*

*Song X-P, Hansen MC, Stehman SV, Potapov PV, Tyukavina A, Vermote EF, et al. Global land change from 1982 to 2016. Nature. 2018; 560:639–43. Epub 2018/08/08.*

*Du L, Niu Z, Zhang R, et al. Evaluation of water resource carrying potential and barrier factors in Gansu Province based on game theory combined weighting and improved TOPSIS model. Ecological Indicators, 2024; 166: 112438.*

*Gao J, O'Neill BC. Mapping global urban land for the 21st century with data-driven simulations and Shared Socioeconomic Pathways. Nat Commun. 2020; 11:2302. Epub 2020/05/08.*

*Wei L, Jin C, Lu Y. Exploring resources and environmental carrying capacities at the county level: A case study of China’s Fengxian County. PloS one, 2019; 14(12): e0225683.*

*Wang S, Fan J, Zhang H, et al. Harmonizing population, grain, and land: Unlocking sustainable land resource management in the farming–Pastoral ecotone. Land, 2023; 12(7): 1311.*

*Zhang H, Zheng J, Hunjra A I, et al. How does urban land use efficiency improve resource and environment carrying capacity?. Socio-Economic Planning Sciences, 2024; 91: 101760.*

*Lu B, Shi Y, Qin S, et al. Evaluating Urban Land Resource Carrying Capacity With Geographically Weighted Principal Component Analysis: A Case Study in Wuhan, China. Transactions in GIS, 2024; 28(7): 2346-2356.*

*Yi Z, Zhou W, Razzaq A, et al. Land resource management and sustainable development: Evidence from China's regional data. Resources Policy, 2023; 84: 103732.*

*Jing X, Tao S, Hu H, et al. Spatio-temporal evaluation of ecological security of cultivated land in China based on DPSIR-entropy weight TOPSIS model and analysis of obstacle factors. Ecological Indicators, 2024; 166: 112579.*

*Ma X, Yuan H. Ecological footprint and carrying capacity of agricultural water-land-energy nexus in China. Ecological Indicators. 2024; 168: 112786.”*

# We deeply appreciate the time and effort these reviewers have dedicated to evaluating our manuscript, and we eagerly await any further feedback or suggestions.
