# Supplementary material for: Spatiotemporal characteristics and optimization strategies of land use and land resource carrying capacity in the three gorges reservoir region (1986–2020)
Source: PLoS One. 2025 Aug 21;20(8):e0330461. doi: 10.1371/journal.pone.0330461 (PMC12370055; doi:10.1371/journal.pone.0330461)
Supplement: Attachment — Submitted filename: Response to Reviewers-R2.docx [file pone.0330461.s003.docx]

Point to Point Responses

**Paper Ref. No.:PONE-D-24-37148R1**

**Title:**Research on the Spatiotemporal Characteristics and Optimization Strategies of Land Use and Land Resource Carrying Capacity in the Three Gorges Reservoir Region over the Past 30 Years.

Thanks so much for all the kind help to this manuscript. We have analyzed the valuable comments from the editor and reviewers#3 carefully, and tried our best to revise the manuscript. We appreciate the thoughtful review and constructive feedback provided by tow viewers.We agree with the reviewer's suggestions and have incorporated the recommended changes into the manuscript.These comments have improved the quality of the paper immensely. The point to point responses are listed as follows.

# Comments（Editor and Reviewer#3）:

Thanks very much for your comments. According to the following valuable comments and suggestions, we have tried our best to revise the manuscript, and these comments have substantially improved the quality of the paper.

**1.Comment:**

In title, the 'research on the' should be deleted, the past 30 years should be revised to specific time span, such as 2000-2023.

**Response:**

Thank you for your suggestion.We have adopted your suggestion by removing the redundant phrasing "Research on the" from the original title and revising "in the Past 30 Years" to "1986-2020". This revision streamlines the title to be more concise and impactful while explicitly highlighting the study's temporal framework (1986-2020), thereby enhancing the precision of academic expression. The specific modifications are outlined below:

*“Spatiotemporal Characteristics and Optimization Strategies of Land Use and Land Resource Carrying Capacity in the Three Gorges Reservoir Region(1986–2020)”*

**2.Comment:**

In abstract, the Landsat images is grid data, while the authors emphasize obtain vector data. In addition, the authors only emphasize the characteristic of spatial and temporal, the influence mechanism is not mentioned. How to optimization strategies without mechanism?

**Response:**

Thank you very much for your valuable comments on our paper. Based on your suggestions, we have revised and improved the abstract. Specifically, we have clarified the nature of Landsat images as raster data and explained how vector data reflecting socio-economic information can be extracted from these raster data. In addition, we have also emphasized the study of the mechanisms behind land use change, exploring how different factors affect the spatial and temporal evolution of land use and carrying capacity, which is crucial for the development of optimization strategies for sustainable development. The specific modifications are outlined below:

*“Studying land use changes caused by human economic activities is beneficial for sustainable growth, making it a global research hotspot. In this study, we used Landsat Thematic Mapper images and statistical yearbooks from 1986, 1995, 2000, 2007, 2010, and 2020 to obtain grid data on the land use status of the Three Gorges Reservoir Region (TGRR), from which vector data reflecting socioeconomic information were derived. We introduced models on land use quantitative changes, dynamic indicators, and degree index to investigate spatiotemporal variations in land use in the TGRR over the past 30 years. Classified maps were generated using ARCGIS 10.8, and Landsat TM images were processed for accuracy using supervised classification techniques. Based on the region's status quo and the analytic hierarchy process, we constructed a land resource carrying ability evaluation indicator model considering social, economic, population, and ecological carrying abilities, introducing a mean-square mistake decision-making approach to determine indicator weights. Our results indicate significant changes in land types within the TGRR from 1986 to 2020, with decreases in arable land, forest land, and grassland, while water bodies, building land, and unused land increased. The change rates varied significantly among different land types, reflecting rapid development, especially between 1995 and 2000. Additionally, our analysis delves into the underlying mechanisms driving these changes, providing insights into how different factors influence spatial-temporal evolution of land use and land carrying capacity, crucial for developing optimization strategies aimed at promoting sustainable growth and efficient use of land resources in the TGRR. This study offers a comprehensive analysis of the TGRR's land resource carrying ability, serving as a reference for sustainable land use.”*

**3.Comment:**

The English should be polished. Previous studies aimed to determine the proportion of people, research on the carrying capacity of land using population and grain. It is difficult to understand. What's meaning of reachesn in line 82.

**Response:**

We thank the reviewers for their valuable comments. The relevant text has been revised and polished in accordance with the requirements, with the typographical error "reachesn" corrected to "reaches" (Line 82). A thorough proofreading and refinement of the entire manuscript has been completed to ensure linguistic accuracy and academic rigor. All modifications have been highlighted in the revised manuscript using track changes for the reviewers' convenience. The specific modifications are outlined below:

*“Land is an important resource necessary for human survival [1–3] . Land use/cover change (LUCC) is the main cause of global climate change and is closely related to human activities. Therefore, studying LUCC has gained global emphasis [4–6]. The carrying ability of land resources is an important index for land resource assessment. Prior research has primarily focused on assessing land carrying capacity, which quantifies the population sustainable by regional food production under multidimensional natural, socioeconomic, and institutional constraints. [7,8].”*

*“The TGRR is a special geographical concept closely related to the Three Gorges Project, and specifically refers to the areas submerged by the construction, storage, and operation of the Yangtze River Three Gorges Project. The TGRR, covering an area of 57,335 km2, is geographically located between 106°20′–110°30 E and 29°–31°50′ E in an area combining the Sichuan Basin and the Middle and Lower Yangtze Valley Plains in the middle and lower reaches (Fig 2). ”*

**4.Comment:**

In line 47-48, why the authors suppose traditional approach for assessing the carrying ability of land is no longer suitable for regional sustainable growth?

**Response:**

Thank you for your valuable suggestion. Regarding your valuable comments on the applicability of traditional land carrying capacity assessment methods, our research team has conducted thorough deliberations and literature re-evaluations. Upon re-examining the discussion in Lines 47-48 of the original manuscript, we confirm that our original intention was to acknowledge the theoretical limitations of conventional approaches in terms of dynamic adaptability and systemic comprehensiveness. Specifically, their unidimensional static assessment framework proves inadequate for characterizing the nonlinear interaction mechanisms within resource-environment-socioeconomic composite systems.

To enhance the academic rigor of this study, we have decided to delete the aforementioned discussion section. The specific modifications are outlined below:

*“With population and financial growth, accelerating urbanization, and intensification of ecological and environmental issues, the demand for land for regional development is constantly expanding. Thus, research on the carrying capacity of land using population and grain as single indicators, is gradually moving towards a comprehensive indicator system [9–12] . ~~The traditional approach for assessing the carrying ability of land is no longer suitable for regional sustainable growth.~~ The existing land carrying ability is the limit of the scale and intensity of different activities that land resources can carry under certain social, economic, and ecological conditions in a certain period and spatial area [9,13].”*

**5.Comment:**

In line 54-56, the authors listed the existing reseach simply. It is lack of summarizing.

**Response:**

Thank you for your valuable suggestion. We sincerely appreciate your constructive feedback regarding the lack of sufficient summarization in our presentation of existing research (lines 54-56). Your insight has prompted our team to engage in thorough discussions and re-examine relevant literature to enhance the synthesis of prior studies. Specifically, we have expanded the section to: (1) systematically categorize key theoretical frameworks and methodological approaches, (2) identify critical knowledge gaps motivating our research. This revised version provides a more comprehensive overview of foundational concepts while clarifying our contribution to advancing this field. Thank you again for your meticulous review - your guidance has significantly strengthened our manuscript. The specific modifications are outlined below:

*“Studying the integrated carrying ability of land resources involves a comprehensive dynamic balance relationship between resources, environment, population, society, economy, and other aspects, thus, reflecting the material, energy, and information flow connections and coordinated development relationships between the natural environment and socioeconomic systems at different regional scales [14–16].~~In 1948, William et al. (1984) proposed that land resource carrying ability is the capacity of land within a region to offer food and shelter for humans and animals, and is being studied abroad since a long time. Feng (1990), one of the earliest scholars in China, developed a land resource carrying ability index based on the association between people and food, and revealed the association between the actual population and land resource carrying ability in the region.~~Land carrying capacity research has undergone a paradigm shift from material supply orientation to system coupling analysis. In the theoretical foundation stage, William (1948) defined land carrying capacity as the basic capacity of a region to support living organisms, Terzaghi (1984) constructed a quantitative assessment framework through a mechanistic model, and Fung's model (1990) created a quantitative research paradigm for the man-food nexus.After the 21st century, Liu Junyan (2010) utilized RS-GIS to reveal the spatial and temporal variations of ecological carrying capacity, and Costanza (1997) expanded the classification of ecosystem services to 17 categories, promoting the diversification of the assessment dimensions. Costanza (1997) expanded the classification of ecosystem services to 17 categories and promoted the diversification of assessment dimensions. In recent years, international research has shown three major frontier advances: first, the deep integration of artificial intelligence and big data, such as the EU LandSense platform (2022), which integrates multi-source data to build a global dynamic monitoring model; second, innovation in system dynamics modeling, the MIT team (2023) proposed the “socioecological-technological” coupled model (SET-CCM), which simulates the evolution of carrying capacity by means of the digital twin technology; and third, the development of the ecological carrying capacity by using RS-GIS technology to reveal the spatial and spatial variability of ecological carrying capacity. Third, interdisciplinary theoretical breakthroughs, the Harvard team (2021) proposed a “planetary boundary carrying capacity” framework, which introduces Earth system science into traditional assessment. At the methodological level, we have broken through the static threshold measurement and developed a dynamic feedback mechanism of “pressure-state-response”, and Stanford University (2024) has constructed a digital twin system for global land carrying capacity, which can simulate the impacts of 200 policy scenarios on resource utilization efficiency. These innovations have significantly expanded the global perspective and prediction accuracy of carrying capacity research, providing scientific quantitative support for sustainable development.”*

**6.Comment:**

In line 57-66, this content could moved to 2.1 study area section, it emphasized the important of taking TGRR as case. This part should introduced existing relevant research and compared the difference with your research.

**Response:**

We deeply appreciate your constructive suggestion regarding the content in lines 57-66. Your feedback has substantially improved our manuscript's clarity and logical flow. Following your guidance, we have relocated the discussion of TGRR's case significance to Section 2.1 to better contextualize our study area within existing research frameworks. Thank you again for your insightful comments - they have demonstrably enhanced the rigor and communication effectiveness of our work..The specific modifications are outlined below:

*“2.1 Overview of the study area*

*The TGRR is a special geographical concept closely related to the Three Gorges Project, and specifically refers to the areas submerged by the construction, storage, and operation of the Yangtze River Three Gorges Project. The TGRR, covering an area of 57,335 km2, is geographically located between 106°20′–110°30 E and 29°–31°50′ E in an area combining the Sichuan Basin and the Middle and Lower Yangtze Valley Plains in the middle and lower reaches (Fig 2). It includes 22 districts and counties, including the central city of Chongqing and four districts and counties, including Yiling District, Yichang City, Hubei Province. The overall terrain of the TGRR is high in the east and low in the west, along with high elevations in both north and south. The terrain is undulating and diverse, with mountains and hills as the main geomorphic features. Mountainous areas account for a large proportion (76.1%), while flat areas account for a small proportion (15.3%). The TGRR area has a subtropical monsoon climate with an annual precipitation of approximately 1000–1800 mm. Owing to the specific original geographical conditions, natural disasters, such as soil erosion, landslides, mudslides, and earthquakes, occur occasionally, among which soil erosion is more severe. Over the past 30 years, as China’s reforms developed and Chongqing was upgraded to the only municipality directly under the central government in the western region, the TGRR has been rapidly developing socioeconomically. Moreover, with the completion and storage of the Three Gorges Project, the land use status and ecological environment have changed significantly. The TGRR has abundant land resources, but the per capita available land is extremely scarce, with the per capita arable land being even less. Thus, the conflict between people and land in this unique region is significant. Since the TGRR has multiple attributes of “super mountainous area” and “super large reservoir area,” it has high research significance.*

*Owing to the unique natural conditions and the establishment of the Three Gorges Project, the ecosystem of the TGRR is unique and fragile that is greatly influenced by its land use status . In the past 30 years, with rapid socioeconomic growth and changes in the natural conditions in the TGRR, the number and spatial features of land use have also changed, leading to significant variations in the land resource carrying capacity [17]. Recently, as ecological migration, urban development, infrastructure construction, and industrial park construction are rapidly increasing in various districts and counties in the TGRR area, land development and construction has consequently increased [16,18–20]. After the water storage capacity of the reservoir area is complete, the carrying ability of land resources changes significantly, which should be systematically studied urgently. Research on the carrying ability of land resources in the TGRR can help to scientifically understand the status of population, resources, environment, and economic growth [5]; alleviate the contradiction between population growth, economic development, ecology, and resources; and drive the sustainable growth of the TGRR.*

*Fig. 2 Location Map of the TGRR.Note:DEM data is sourced from the Geospatial Data Cloud website (http://www.gscloud.cn/home).Administrative boundary data comes from Resource and Environment Science and Data Center (https://www.resdc.cn/),and the map boundary has not been changed.Cartographic software:ARCGIS 10.8.”*

**7.Comment:**

In line 73, spatiotemporal is not novelties. The novelties including perspective, method or indicators.

**Response:**

We are grateful for your insightful feedback regarding our presentation of novel contributions in line 73. Your observation that "spatiotemporal" alone does not constitute methodological novelty has prompted us to re-examine and refine our discussion of research innovations. We fully agree that true novel contributions should be framed through original theoretical perspectives, methodological frameworks, or indicator systems. Accordingly, we have revised this section..The specific modifications are outlined below:

*“Analytic hierarchy process (AHP) was introduced to build an assessment indicator model for the land resource carrying capacity from several dimensions, such as social, economic, population, and ecological carrying abilities. ~~Simultaneously, the weight of the index was determined using a mean square error decision-making approach. Studying the spatiotemporal characteristics of land use change patterns and land resource carrying capacity in the TGRR offers certain novelties in research perspectives, spatiotemporal dimensions, and other aspects. The framework diagram of the research thinking in this study is presented in Fig 1.~~Studying the spatiotemporal characteristics of land use change patterns and land resource carrying capacity in the TGRR provides novel contributions in research perspectives, methodological approaches, and indicators. The framework diagram of the research methodology is presented in Fig 1.”*

**8.Comment:**

The framework diagram should be move to method section, and illustrate each of steps clearly.

**Response:**

Thank you very much for your valuable comments on our paper. Regarding your suggestion of moving the framework diagram to the methodology section and clearly showing each step, we have revised and improved the paper accordingly based on your feedback. Specifically, we have adjusted the framework diagram to the methodology section and elaborated the specific content and implementation details of each step in the text to ensure that the whole assessment process is clearer and easier to understand..The specific modifications are outlined below:

*“The study adopts a systematic framework for assessing land carrying capacity, which is divided into the following three main steps: in the first step, a three-level indicator system containing four first-level indicators (demographic, economic, social, and ecological carrying capacity) is constructed by acquiring and pre-processing data in the study area (including determining the scope, unifying the resolution of the data, and extracting the land-use information); in the second step, the current land-use situation is analyzed, the Calculate the standard values of land use changes (e.g., K, Lc, L) using specific formulas, and classify and quantify the land use data in order to clarify the changes in land use; in the third step, at the stage of comprehensive land carrying capacity assessment, standardize the indicators of the same guideline layer through the maximum-minimum value method, then apply the hierarchical analysis method (AHP) to determine the weights of each evaluation indicator, and finally, based on the The standardized indicator values and weights are then used to determine the weights of each evaluation indicator, and finally the integrated evaluation value (Di(W)) is calculated based on the standardized indicator values and weights to arrive at the final conclusion about the land carrying capacity. This framework provides a comprehensive assessment of the land carrying capacity of the study area through a multi-step systematic analysis. The framework diagram of the research methodology is presented in Fig 1.*

*Fig. 2 Research framework diagram.Note:The basemap was obtained from the Geospatial Data Cloud(http://www.gscloud.cn/home), and the map boundary has not been changed.Cartographic software:ArcGIS 10.8.”*

**9.Comment:**

The content of 2.1 is basic information of study area, it is useless.

**Response:**

Thank you for your valuable comments on the manuscript. Regarding the content of section 2.1 (Overview of the study area), we have fully considered your suggestions and have streamlined and optimized it by removing redundant background descriptions and focusing on key information directly related to this study, such as the unique geography, hydrological characteristics, and socioeconomic attributes of the Three Gorges Reservoir Area. We retained this section because the Three Gorges Reservoir Area, as one of the largest and most ecologically complex water conservancy project impact areas in the world, has a special geographic location, ecological sensitivity, and human-land relationships that are important to support the study's conclusions. Considering that international readers may lack systematic knowledge of the region, a modest background introduction helps to enhance the logical coherence of the study and the interpretability of the conclusions. The revised text has further emphasized its relevance to the theme of the study, and we thank you for your review and correction! The specific modifications are outlined below:

*“The TGRR is a special geographical concept closely related to the Three Gorges Project, and specifically refers to the areas submerged by the construction, storage, and operation of the Yangtze River Three Gorges Project. The TGRR, covering an area of 57,335 km^2^, is geographically located between 106°20′–110°30 E and 29°–31°50′ E in an area combining the Sichuan Basin and the Middle and Lower Yangtze Valley Plains in the middle and lower reaches (Fig 1). The Three Gorges Reservoir Region (TGRR), spanning 22 districts and counties including Chongqing's core urban area, is characterized by a mountainous-dominated geomorphology (76.1% mountainous terrain, 15.3% flatlands) with distinct elevation gradients and a subtropical monsoon climate (annual precipitation: 1000–1800 mm). Its dual identity as a "super mountainous" and "super reservoir" zone exacerbates ecological vulnerabilities, particularly severe soil erosion and landslide risks linked to steep slopes and hydrological fluctuations. Since 1986, accelerated socioeconomic growth driven by Chongqing's municipal status elevation and the Three Gorges Project's impoundment has triggered dramatic land use transitions, intensifying the paradox between abundant total land resources and acute per capita scarcity (notably arable land <0.05 ha/person). These spatiotemporal dynamics, shaped by reservoir inundation, terrain constraints, and anthropogenic pressures, position the TGRR as a critical nexus for analyzing land resource carrying capacity evolution and formulating adaptive optimization strategies under coupled ecological and developmental stresses.”*

**10.Comment:**

In 2.2 data sources, it should be introduced more detailed, such as website, resolution etc.

**Response:**

Thank you for your careful review and valuable suggestions on the data source section. After careful verification and supplementation, we have further improved the details of the data descriptions in Section 2.2, including: (1) clearly labeling the download platforms of the remote sensing image data (e.g., Google Earth Engine); and (2) adding the spatial resolution (e.g., 30 meters for Landsat TM) and temporal coverage of the key data. These additions are intended to enhance the transparency and reproducibility of the study, and to ensure that readers can clearly trace the data sources and technical processes. Thank you again for your expertise. The specific modifications are outlined below:

*“2.2 Data sources*

*In this study, Landsat Thematic Mapper (LTM) false-color remote sensing image data (https://search.earthdata.nasa.gov) with an accuracy of 30m*30m were used. administrative division data were obtained from the National Geographic Information Center of China (http://www.ngcc. cn/ngcc/). In addition, population, economic, social, and ecological data were converted from Chongqing Statistical Yearbook and Hubei Provincial Statistical Yearbook (1986-2020) and related data such as land use. Land use types were categorized by supervised classification method, and the accuracy comparison results based on 688 randomly selected ground truth sample points showed that the accuracy was 94.52%. Comparison of the accuracy based on 688 randomly selected ground-truth sample points shows that the accuracy is 94.52% and the Kappa coefficients are also highly consistent, thus confirming the accuracy and scientific value of the data.”*

**11.Comment:**

In section 2.3.1, this method is too simply, it is not necessary to shown.

**Response:**

Thank you for your review and advice on the Methods section of the paper. We have carefully evaluated and adopted your comments on the abbreviation of section 2.3.1, and decided to delete this section to avoid redundancy and improve the conciseness and readability of the whole paper. The revised manuscript further focuses on the elaboration of the core research methodology to ensure that the technical line is clear and logically coherent. Your suggestions have played an important role in optimizing the structure of the paper. Thank you again for your professional guidance. The specific modifications are outlined below:

“*~~2.3.1 Quantitative Changes in Land Use~~*

*~~Studies of the characteristics of land use changes have focused on spatiotemporal variations in land use, including variations in the quantity, spatial distribution, and degree of land use [7,21–23] . To understand the laws of land use variations, land use status data of the TGRR in 1986, 1995, 2000, 2007, 2010, and 2020 were classified and statistically analyzed spatiotemporally using RS and GIS.It's worth noting that, due to limitations in the availability of satellite data, interpolation of land use data was required for certain periods, which may introduce some minor uncertainties in the analysis.~~*”

**12.Comment:**

The authors used land use index model to classify land use categories? It is blur. What's the meaning of land use degree? Maybe it is category or type. The degree means high or low.

**Response:**

Thank you for your interest and valuable comments on the Land Use Extent Index model! The issues you raised are very important and we have further clarified the conceptual and methodological details in the revised version. Your comments have motivated us to present the methodological details more rigorously, and we appreciate your help in improving the clarity of the paper. The specific modifications are outlined below:

*“2.3.2 Land Use Index Model*

*The breadth and depth of land use are mainly reflected in the degree of land use, the natural attributes of land, and the degree of influence of human activities on land [24-26]. Based on the comprehensive analysis method of land use level proposed by Liu et al. and combining with the current situation of TGRR, we divide the land use level into four levels to obtain the grading index of land use degree (Table 1). In this context, “land use degree” refers to the intensity and scope of land development and use, rather than a simple category or type, which reflects the high or low degree of land use. Subsequently, we calculated the land use level index through equation (3) to quantitatively assess the land use in the study area. Such an approach not only refined the land use classification, but also enabled us to understand the impact of different land use patterns more precisely..*

*Table1 Classification of land use index.”*

**13.Comment:**

In line 141-142, the indicators are in 2.3.4, which indicators in 2.3.3 refer to ? It is lack of logic.

**Response:**

Thank you for your interest in the methodology for evaluating the carrying capacity of land resources! The clarity of the indicators and weights you mentioned is very important. We have further optimized the relevant expressions in the revised draft, and clearly marked the specific table (i.e. “indicators (Table 2)”) after “indicators”, so that readers can directly compare the contents of the table. The specific modifications are outlined below:

*“where I_a_ and I_b_ regional land use indices represent the initial and termination periods, respectively, Ai represents the classification index of the extent of i-level land use, and Ci_b_ and Ci_a_ represent the extent of i-level land use during the initial and terminal study periods, respectively. This formula can effectively calculate the degree of land use change in the study area.The selection of indicators(Table 2)for the land resource carrying capacity was based on an analytic hierarchy process (AHP), with weights determined using a mean-square decision-making approach. Each criterion was verified by experts in the field for robustness.”*

**14.Comment:**

Is it AHP method in 2.3.4? It is not necessary to describe it too complex. The weight of indicators are not illustrated.

**Response:**

Thank you for your careful review of the methodology description. Regarding the description of AHP (hierarchical analysis method) in section 2.3.4, we have streamlined the content according to your suggestions, deleted redundant technical details, and retained only the overview of the core process (e.g., the construction of the judgment matrix and consistency test). The specific calculation process and results of the indicator weights have been adjusted to be systematically described in section 3.5.2 “Determination of Indicator Weights”, and the weights of the indicators at each level are presented in Table 8. The purpose of this adjustment is to more clearly separate the methodological framework from the application of weights and to avoid duplication of information between sections. If you think there is still a need for further simplification or additional explanation, we will improve it according to your guidance. Thank you again for your valuable comments on optimizing the structure of the paper. The specific modifications are outlined below:

*“2.3.5 Evaluation Method for the Land Resource Carrying Capacity*

*In the multi-indicator comprehensive evaluation method, weight coefficients of attribute indicators can be determined by subjective weighting approaches (e.g., Gulin, Delphi, and AHP) or objective weighting approaches (e.g., principal component analysis, element analysis, and mean square error method) [29,30]. While subjective methods are widely applied, their results may lack objectivity due to human bias. In contrast, objective methods like the mean square error approach offer higher clarity, computational simplicity, and accuracy in weight determination [31].*

*Considering the practical context of the Three Gorges Reservoir Region (TGRR), we adopted the mean square deviation approach. This method treats each assessment indicator as a random variable, with dimensionless values of schemes under each indicator as variable realizations. First, the mean square deviation of each indicator is calculated and normalized to derive its weight. The standardized indicator values are then multiplied by their respective weights to obtain subsystem evaluation values (e.g., bearing subsystems), and summed to generate the comprehensive land-bearing capacity score.*

*First, the mean E (Aj) of the random variable is calculated as: ”*

**15.Comment:**

In 2.3.5, it is only standardization, it is not necessary to introduced independent. It is a progress of AHP.

**Response:**

Thank you for your careful review and suggestions on the methodology section! Regarding the standardization process in subsection 2.3.5, we have revised it according to your comments by deleting the standardization step in the original section 2.3.5, and clarifying it as a pre-data processing link for AHP weight calculation to avoid redundancy. The specific modifications are outlined below:

### *“***~~2.3.4 Standardization of Evaluation Indicators~~**

~~Given that the assessment of land resource carrying ability involves several domains, has multiple indicators, and the measurement units of every evaluation index are inconsistent, they cannot be used directly to assess the land resource carrying ability. Therefore, before analyzing the data in terms of various evaluation indicators, they should be standardized. Moreover, to eliminate the impact of dimensionality on the evaluation results, dimensionless processing on the data of each indicator should be performed. This study used the range transformation method [28] to perform dimensionless standardization of indicators, and the processing approach was as follows:~~

~~For positive/moderate indicators,~~

 ~~(1 ≤ i ≤ n, 1 ≤ j ≤ m)	(6)~~

~~For reverse indicators,~~

 ~~(1 ≤ i ≤ n, 1 ≤ j ≤ m)	(7)~~

~~where i is the time, j is the evaluation indicator, and yij is the dimensionless value of time i and time j evaluation indicator. Further, Xij represents raw data for the ith time of the assessment indicator j, maxXij and minXij are the maximum and minimum values of the i-time j evaluation index, with a standardized value of 0 ≤ yij ≤ 1 [9,24].~~*”*

**16.Comment:**

In section 2.3.6，this is also AHP, an AHP is very simple and doesn't have to be broken down into many methods,It's too messy. You need to explain how many methods are used in this article and what problems each method solves.

**Response:**

Thank you for your valuable comments on the Methods section! We fully agree with your suggestion that the logic of the methodology needs to be concise and clear, and have fully restructured and integrated the chapter structure. The specific modifications are as follows: “2.3.1 Land use dynamics model (to quantify the rate of land use change); 2.3.2 Land use composite index model (to assess the degree of land use and spatial differentiation); 2.3.3 Land resources carrying capacity evaluation index system (to construct multi-dimensional indexes and weights based on the AHP); 2.3.4 Evaluation method of land resources carrying capacity (to integrate the mean-variance method to calculate the comprehensive score). (integrating the mean-variance method to calculate the comprehensive score).”

**17.Comment:**

The results is too simple. The authors divided a problem into several parts. The results should contain three problems. First, the temporal and spatial evolutuion of land use, the spatial agglomeration characteristics, and the change of growth rate. Secondly,the temporal and spatial evolution characteristics of land carrying capacity. Thirdly, the impact of land use changes on land carrying capacity.

**Response:**

Thank you for your careful review of the results section and your valuable suggestions! We fully agree with your proposed integration of the framework and deepen the direction of the analysis of the guiding significance, and has been strictly in accordance with your comments on the conclusion of the part of a comprehensive revision of the revised conclusions to the “evolution of land use - changes in carrying capacity - impact analysis” as the main line, delete redundant expressions and integrate data logic to ensure that the level of clarity and focus of the issue. The revised conclusion is based on the line of “Land use evolution - Carrying capacity change - Impact analysis”, deleting redundant expressions and integrating the logic of data to ensure the clarity of the hierarchy and focus of the problem. Your professional advice has greatly enhanced the rigor and depth of the study. Thank you again for your guidance. The specific modifications are as follows:

*“This study used RS and GIS technologies to systematically analyze the spatiotemporal characteristics of quantitative changes in the land use type, speed of land use type changes, and land use degree in the TGRR from 1986 to 2020, using models related to land use variation. According to the indicator system of the “population–society–economy–ecology” dimension and using the carrying ability model, the land resource carrying ability was comprehensively evaluated, and the following conclusions were obtained:*

*(1) Between 1986 and 2020, land use types in the TGRR were mainly forest land and cropland. In particular, the area of built-up land and watersheds continued to increase, while the area of grassland, cropland and woodland decreased. In particular, the fastest increase was in construction land and the slowest change was in forest land. The composite degree index of land use types showed an increasing trend for all types of land use, indicating that the region was in a state of development during the study period, and that the fastest rate of land use development was observed during the period 1995-2000. In addition, the rate and process of change of different land use types showed diversity.*

*(2) From 1986 to 2020, the carrying capacity of land resources in the TGRR, including demographic, economic, social and ecological carrying capacity, generally increased, especially the ecological carrying capacity reached a high level. The comprehensive carrying capacity of land resources has gradually increased from weak to strong, and overall maintained a relatively stable upward trend without significant fluctuations. Especially during the period 1986-2010, the growth rate of the comprehensive carrying capacity was relatively slow, while in the subsequent period, the overall carrying capacity of land resources in the TGRR showed a rapid upward trend.*

*(3) During the study period, the land resources, demographic, economic, social and ecological factors of the TGRR have changed significantly, and the whole process of change has been more complicated. With the continuous increase in the area of construction land and watershed, and the decrease in the area of cultivated land, grassland and forest land, these changes have directly affected the carrying capacity of land. Specifically, this is manifested in the substantial increase in the carrying capacity of economic, social and ecological systems, especially the ecological carrying capacity is at a high level, occupying the largest proportion of the comprehensive carrying capacity of land resources in the TGRR. This indicates that the adjustment of land use structure is of great significance in enhancing the comprehensive carrying capacity of land and promoting sustainable development.*

*~~(4) During 1986–2020, land resources, population, economy, society, and ecological factors changed significantly in the TGRR, and the process of change was relatively complex. The population, economic, social, and ecological carrying abilities of land resources were generally increasing; particularly, the ecological carrying capacity was high.~~*

*~~(5) From 1986 to 2020, the integrated carrying ability of land resources in the TGRR increased from weak to high, and its overall upward trend was relatively stable without significant fluctuations. During 1986–2010, the growth rate of the integrated carrying ability of land resources increased relatively slowly, while the overall carrying ability of land resources in the TGRR displayed a rapid upward trend in the remaining period. The economic, social, and ecological carrying capacities within the system greatly improved, with the ecological environmental carrying capacity at a relatively high level, accounting for the highest proportion of the integrated carrying ability of land resources in the TGRR.~~*

*Overall, the results of this study are objective and authentic, and the construction of evaluation indicators can adapt to the regional and development characteristics of the TGRR. The evaluation method fully reflects the differences in land use changes and comprehensive carrying capacity development in different periods and regions of the TGRR, and reflects the contradictory relationship between land resources, ecological environment, and regional social and economic development. Finally, this study has certain reference significance and value in both the construction of evaluation indicators and the selection of evaluation methods in future land bearing capacity research.”*

# We deeply appreciate the time and effort these reviewers have dedicated to evaluating our manuscript, and we eagerly await any further feedback or suggestions.
