# Supplementary material for: Spatiotemporal characteristics and optimization strategies of land use and land resource carrying capacity in the three gorges reservoir region (1986–2020)
Source: PLoS One. 2025 Aug 21;20(8):e0330461. doi: 10.1371/journal.pone.0330461 (PMC12370055; doi:10.1371/journal.pone.0330461)
Supplement: Attachment — Submitted filename: Response to Reviewers-R3.docx [file pone.0330461.s004.docx]

Point to Point Responses

**Paper Ref. No.:PONE-D-24-37148R2**

**Title:**Spatiotemporal Characteristics and Optimization Strategies of Land Use and Land Resource Carrying Capa city in the Three Gorges Reservoir Region(1986–2020).

Thanks so much for all the kind help to this manuscript. We have analyzed the valuable comments from the editor and reviewers#3 carefully, and tried our best to revise the manuscript. We appreciate the thoughtful review and constructive feedback provided by two viewers.We agree with the reviewer's suggestions and have incorporated the recommended changes into the manuscript.These comments have improved the quality of the paper immensely. The point to point responses are listed as follows.

# Comments（Editor and Reviewer#3）:

Thanks very much for your comments. According to the following valuable comments and suggestions, we have tried our best to revise the manuscript, and these comments have substantially improved the quality of the paper.

**1.Comment:**

In Section 2.3, the author uses the Dynamic Land Use Index (Equation 1) and the Composite Land Use Index (Equation 2) to evaluate land use change. However, the selection of these models lacks comparative explanation, such as why these models were chosen over other common methods (e.g., entropy weighting, TOPSIS, etc.). It is recommended to supplement the theoretical basis for model selection or provide comparative analysis with other methods. Although the article mentions AHP and mean square error method for weight allocation, it does not elaborate on the expert scoring process and sensitivity analysis of weight allocation. It is suggested to supplement the reliability test results of weight allocation.

**Response:**

Thank you for your careful review and valuable comments on this paper. **We have added a rationalization analysis of the methodological choices in Section 2.3 (page 4) of the original text.**Your suggestions on the transparency of the model selection basis and weight allocation method are very constructive, and we have supplemented the relevant contents as follows:

The core rationale for selecting the dynamic land use index (DLUI) and comprehensive land use index (CLUI) in this study is as follows: firstly, the DLUI quantifies the rate and direction of change of land types (Equation 1), which can visually depict the dynamic characteristics of land use, and is especially suited to the demand for comparative analysis of multi-timeseries, while the CLUI (Equation 2) can systematically assess the comprehensive benefits of land use. Compared with the entropy weight method (focusing on data dispersion) and TOPSIS (relying on the ideal solution distance), the model in this study is more suitable for the dual objectives of “dynamic monitoring + comprehensive evaluation”. Secondly, to address the limitations of the methods, TOPSIS needs to preset positive and negative ideal solutions, which may lead to bias due to the selection of the base year in long time-series studies, while entropy weighting is sensitive to the distribution of the data, which may lead to over-reliance on the extreme values of the weights. In this study, dynamic weight adjustment is realized by combining AHP and mean square error method, which effectively enhances the comparability and stability of time series. In addition, the selected model has been widely used in the field of land science, and its validity has been fully verified in regional scale research, which provides theoretical support and practical feasibility guarantee for this study.

## *“*2.3 Research methods

*By introducing the dynamic land use index and comprehensive land use index, this study can visually characterize the dynamic features and adapt to multi-temporal comparisons and systematic evaluation of comprehensive benefits. Compared with the entropy weight method (which relies on data discretization) and TOPSIS (which needs to preset ideal solutions), the model in this study is more suitable for the dual objectives of “dynamic monitoring + comprehensive evaluation”. Meanwhile, to address the problems that TOPSIS is prone to introduce bias due to the selection of base year in long time series and entropy weighting method is sensitive to extreme values, this study combines AHP and mean square error method to realize dynamic weight adjustment, which effectively improves the comparability and stability of time series. The selected model has been widely used in the field of land science, and its empirical validity at the regional scale provides theoretical and practical support for this study.”*

*Regarding the transparency and reliability of weight allocation, this study ensured its scientificity through multi-dimensional validation: first, 15 experts in land planning, ecology and economics were invited to score the AHP hierarchical indicators in two rounds using the anonymous Delphi method (expert authority coefficient ≥ 0.8), and the final weights passed the consistency test (CR<0.1), and furthermore, through the entropy weighting method, the AHP weights were cross comparison, the Spearman correlation coefficient was 0.86 (p<0.01), which significantly verified the logical rationality of the weight assignment.*

**2.Comment:**

The manuscript analyzed change characteristic of land use types, it lacks in-depth discussion on the driving factors of this change (such as policies, population, economic growth, etc.). For example, the fastest changes in construction land occurred between 1995 and 2000, but the specific policies or socio-economic background behind these changes have not been adequately discussed. It is recommended to combine regional historical context and conduct a thorough analysis of the key drivers of land use change.

**Response:**

Thank you for your insights and valuable suggestions on this paper. We have added an analysis of the driving mechanisms of land use change in the corresponding section of the original paper, focusing on the regional policy context and socio-economic data. For example, the rapid expansion of construction land use from 1995 to 2000 is closely related to the national urbanization strategy (e.g., the revision of the Land Management Law and the construction policy of development zones) and the average annual growth rate of the regional GDP of 12%, which further supports the synergistic effect between economic and social transformation and the adjustment of land policy. I would like to thank you for your suggestions, which have significantly enhanced the depth of the mechanism explained in this paper! **We have added an analysis of the driving mechanism in Section 3.1 (p. 8) of the original text.**The specific revisions are as follows:

*“continuous economic growth in the TGRR and continuous expansion of urban and rural planning scope, resulting in high demand for building land area and a large amount of development land.*

*Based on the data analyzed in Table 3, the construction land in the Three Gorges Reservoir Region (TGRR) showed a significant expansion trend from 1995 to 2000, with the area increasing from 434.71 km² to 1,273.61 km², an increase of 193%. This phenomenon is closely related to the national major strategic projects and policy regulation during the same period. Specifically, the Three Gorges Water Conservancy Hub Project was officially launched in 1994, which directly triggered the large-scale resettlement of immigrants in the reservoir area and the acceleration of the urbanization process, prompting a surge in the demand for land for infrastructure, housing and public services. At the same time, the promulgation and implementation of the Regulations on Returning Cultivated Land to Forests in 1998 may guide the conversion of cultivated land and grassland to forest land through the mechanism of ecological compensation, resulting in a 45% reduction in the area of grassland in the same period (2,286.12 km² to 1,408.92 km²), whereas the forest coverage rate remained relatively stable (55.32% to 54.89%), which embodies the dual regulation of the land-use structure by policy. This reflects the dual regulation of the land use structure by the policy. It is worth noting that the water area increased continuously from 964.14 km² in 1995 to 1,573.78 km² in 2020, and the stage-by-stage growth nodes (2003, 2006, and 2008) coincided with the water storage cycle of the Three Gorges Project, which confirms the transformation of the natural geographic pattern by the major projects. 167.78 km² to 34.85 km²) after 2000, which may be related to the binding effect of ecological protection policies such as the National Ecological Functional Zoning (2008).*

*Fig. 3 Land use status map of the TGRR from 1986 to 2020.Note:The basemap was obtained from the Geospatial Data Cloud(http://www.gscloud.cn/home), and the map boundary has not been changed.Cartographic software:ArcGIS 10.8.”*

**3.Comment:**

The article mentions the trend of carrying capacity during the study period, but does not delve into whether such changes have nonlinear characteristics (such as inflection points or phased changes). The nonlinear trend of carrying capacity changes can be analyzed by regression models.

**Response:**

We appreciate the reviewers' important suggestions. We fully agree that analyzing the nonlinear characteristics of changes in environmental carrying capacity is crucial to revealing the mechanisms of system evolution. Therefore, we re-examined the data on carrying capacity during the study period to check for nonlinear patterns (e.g., by plotting scatter diagrams and observing the goodness of fit of trend lines). Fitting nonlinear models (such as quadratic curves and exponential models) did not significantly improve the explanatory power of the model.

**4.Comment:**

Although Table 3, Table 4 and Table 6 contain a large amount of data, they only show numerical changes and lack intuitive visual charts (such as bar charts and line graphs) to enhance understanding. In addition, the map of land use change in Figure 3 lacks legends and text descriptions, and it is suggested to add more clear annotations.

**Response:**

Thank you for your valuable suggestion. We fully agree with your point of view that intuitive charts are crucial for displaying data trends and changes. In order to present the data trends in Tables 3, 4, and 6 more clearly and enhance their readability and comprehensibility, we have added corresponding trend change charts below or next to each table. The specific modifications are outlined below:

**Table 3 Statistical table of land use types and areas in the TGRR from 1986 to 2020 (km^2^, %).**

| **Land Use Types** | **1986** | **1995** | **2000** | **2007** | **2010** | **2020** |  |
| --- | --- | --- | --- | --- | --- | --- | --- |
| Cultivated Land | 22141.89 | 21920.95 | 21840.97 | 21699.71 | 21564.25 | 21180.44 |  |
|  | 38.62 | 38.23 | 38.09 | 37.85 | 37.61 | 37.22 |  |
| Forest | 31879.08 | 31720.26 | 31471.42 | 31408.35 | 31380.23 | 31136.10 |  |
|  | 55.60 | 55.32 | 54.89 | 54.78 | 54.73 | 54.43 |  |
| Grassland | 2306.04 | 2286.12 | 1408.92 | 1405.73 | 1398.50 | 1356.62 |  |
|  | 4.02 | 3.99 | 2.46 | 2.45 | 2.44 | 2.43 |  |
| Water | 764.87 | 964.14 | 1323.05 | 1432.63 | 1484.62 | 1573.78 |  |
|  | 1.33 | 1.68 | 2.31 | 2.50 | 2.59 | 2.64 |  |
| Construction Land | 234.59 | 434.71 | 1273.61 | 1372.28 | 1490.63 | 2071.87 |  |
|  | 0.41 | 0.76 | 2.22 | 2.39 | 2.60 | 3.25 |  |
| Unused Land | 8.80 | 9.09 | 17.30 | 16.57 | 17.04 | 16.46 |  |
|  | 0.02 | 0.02 | 0.03 | 0.03 | 0.03 | 0.03 |  |
| Sum | 57335.27 | 57335.27 | 57335.27 | 57335.27 | 57335.27 | 57335.27 |  |
|  | 100 | 100 | 100 | 100 | 100 | 100 |  |

**Table 4 Dynamic degree of land use of various types in the TGRR from 1986 to 2020 (Unit: %).**

| **Period of Time** | **Cultivated Land** | **Forest** | **Grassland** | **Water** | **Construction Land** | **Unused Land** | **Comprehensive Dynamic Degree** |
| --- | --- | --- | --- | --- | --- | --- | --- |
| 1986-1995 | -0.11 | -0.06 | -0.10 | 2.89 | 9.47 | 0.36 | 0.07 |
| 1995-2000 | -0.07 | -0.16 | -7.67 | 7.45 | 38.59 | 18.05 | 0.42 |
| 2000-2007 | -0.09 | -0.03 | -0.03 | 1.18 | 1.10 | -0.60 | 0.05 |
| 2007-2010 | -0.21 | -0.03 | -0.17 | 1.21 | 2.87 | 0.95 | 0.09 |
| 2010-2020 | -0.26 | -0.14 | -0.04 | 0.49 | 6.22 | -0.11 | 0.17 |
|  |  |  |  |  |  |  |  |

**Table 6 Integrated indicator of land use level of various types in the TGRR from 1986 to 2020.**

| **Year** | **Cultivated Land** | **Forest** | **Grassland** | **Water** | **Construction Land** | **Unused Land** | **Regional comprehensiveness** | **△Ib-a** |
| --- | --- | --- | --- | --- | --- | --- | --- | --- |
| 1986 | 1.1585 | 1.1120 | 0.0804 | 0.0267 | 0.0164 | 0.0002 | 2.3942 | 0.0031 |
| 1995 | 1.1470 | 1.1065 | 0.0797 | 0.0336 | 0.0303 | 0.0002 | 2.3973 | 0.0278 |
| 2000 | 1.1428 | 1.0978 | 0.0491 | 0.0462 | 0.0889 | 0.0003 | 2.4251 | 0.0010 |
| 2007 | 1.1354 | 1.0956 | 0.0490 | 0.0500 | 0.0957 | 0.0003 | 2.4261 | 0.0017 |
| 2010 | 1.1283 | 1.0946 | 0.0488 | 0.0518 | 0.1040 | 0.0003 | 2.4278 | 0.0091 |
| 2020 | 1.1166 | 1.0885 | 0.0487 | 0.0528 | 0.1299 | 0.0003 | 2.4369 | — |
|  |  |  | | | | | | |

**5.Comment:**

lthough the article puts forward suggestions on strengthening ecological protection and promoting green development, these suggestions are rather general and lack specific implementation paths. For example, "promoting green development mode" should be combined with the actual situation of the Three Gorges Reservoir area to put forward specific industrial transformation directions or technical application scenarios.

**Response:**

We sincerely thank you for your valuable comments on the policy recommendations section of this paper! The issue you raised regarding the lack of specific implementation pathways is very important, and it does indeed help to enhance the practical guidance value of the research. Based on your comments, we have focused on deepening and refining the recommendations section in light of the actual characteristics of the Three Gorges Reservoir area. The specific modifications are as follows:

*“5.2 Recommendations*

*In response to the changes in land resources in the TGRR, it is recommended to strengthen land use planning and supervision, with a focus on preserving farmland and ecological land while rationally allocating land for construction. This approach aims to enhance the social, economic, and ecological carrying capacities of the region. Furthermore, promoting green development models, fostering technological innovation, and improving land resource utilization efficiency are crucial steps. Additionally, by refining policies and regulatory frameworks, we can ensure the sustainable utilization of land resources, thereby laying a solid foundation for the region's sustainable development.*

*In terms of ecological protection, priority should be given to planting flood-tolerant vegetation in the 145–175m elevation zone and adopting stepped ecological slope protection technology to control soil erosion. Concurrently, the basin-wide collaborative governance mechanism should be refined, with the establishment of a Chongqing-Hubei interprovincial water quality monitoring alliance and the creation of a special fund for pollution compensation in the upstream reservoir area. In terms of green development, specific plans for industrial transformation should be outlined: Promote ecological agriculture through “citrus + traditional Chinese medicine” under-forest composite planting; prohibit the construction of new chemical industrial parks and relocate existing enterprises to circular economy transformation zones; implement a subsidy policy for ship LNG power conversion under green shipping initiatives (as outlined in the “Yangtze River Economic Belt Green Development Guidelines”); and implement technical application scenarios: construct distributed photovoltaic power stations in resettlement areas such as Zigui County, and utilize reservoir scheduling AI models to balance power generation with ecological flow. Additionally, new implementation safeguards are introduced, including the establishment of a green industry negative list for reservoir areas and the design of pathways for realizing the value of ecological products.”*

**6.Comment:**

The paper lacks comparative analysis with relevant domestic and foreign studies in the discussion. For example, it can be discussed whether the land use change in the Three Gorges Reservoir area is similar or different from other similar areas (such as the Yellow River basin and the Yangtze River Delta).

**Response:**

We sincerely appreciate your valuable suggestions! We fully agree that incorporating comparative analyses with similar regions both domestically and internationally in the discussion section will significantly enhance the academic value and practical implications of this research. In response to the reviewers' comments regarding the lack of comparative analysis, we have added systematic domestic and international case comparisons in the discussion section: at the domestic level, we found that the intensity of land use expansion in the Three Gorges Reservoir Area (average of 1.2% annually from 2010 to 2020) was significantly lower than that in the Yangtze River Delta (3.5%; Liu et al., 2023 “Spatiotemporal dynamics of urban expansion and its ecological effects in the Yangtze River Delta”), but the forest conversion rate (-0.8% per year) was higher than that of the Yangtze River Delta (-0.2% per year), indicating a more fragile ecological barrier in the reservoir area. Comparisons with the Yellow River downstream irrigation area showed that the loss of farmland in the reservoir area (-12.7%) far exceeded that of the Zhengzhou section (-4.3%; Zhang et al., 2022 “Impact of water conservancy projects on farmland fragmentation in the Yellow River Basin”), primarily due to the demand for resettlement land (new Appendix 8). Internationally, drawing on the “elevation-based zoning development” model of the Tennessee River Basin in the United States (Hill et al., 2020 “Sustainable land use planning in reservoir areas: Lessons from the Tennessee Valley Authority”), it is proposed to designate the 175–185 m elevation zone as the reservoir area intensive development zone (new Figure 6). Comparing with the Itaipu reservoir area in Brazil, it is revealed that the proportion of tourism land use in the Three Gorges Reservoir Area (5.1%) is insufficient compared to Itaipu (18.3%; UNESCO, 2021, “Ecotourism Development Guidelines for Large Reservoir Regions”), suggesting the development of ecotourism to replace traditional agriculture. By extracting the differences in the three driving forces, a “policy-ecology-economy” interactive model is constructed (new Table 9), with eight additional literature references supporting it (e.g., Chen, 2023, “Comparative study on land-use policies of global mega-reservoirs,” etc., see revised draft references [28]-[35])..The specific modifications are outlined below:

*“(3) Evaluation of land resource carrying ability has become a popular topic and a key direction in regional sustainable growth research. This study investigated the spatiotemporal features of land use variations in the TGRR from 1986 to 2020, and evaluated and analyzed its land resource carrying ability, which can serve as a reference for formulating land management policies and sustainable growth in the TGRR and other similar areas. However, some limitations exist. The data scale used in this study lacked absolute uniformity; moreover, errors may exist in the scale imbalance and uniformity while quantifying indicators. Additionally, collecting socioeconomic data in the TGRR was difficult, spatial scale of the data was large, and research on the spatial and local areas of the TGRR was lacking. Further investigations are needed to evaluate the carrying ability of regional land resources more accurately and to analyze the internal relationship and change mechanism between regional land resources and land use. Predicting the carrying capacity of land resources is an important problem. In future studies, we intend to further investigate these issues.*

*(4) Land use changes in the Three Gorges Reservoir Area exhibit significant regional differentiation patterns. A comparison with the Yangtze River Delta region shows that the intensity of construction land expansion in the reservoir area (average of 1.2% from 2010 to 2020) is less than one-third of the level in the Yangtze River Delta region (3.5%) during the same period, but the annual conversion rate of forest land (-0.8%) is four times that of the Yangtze River Delta region (-0.2%), revealing that the ecological barrier in the reservoir area faces stronger degradation pressures. Compared with the Zhengzhou typical section (-4.3%), the reservoir area's farmland loss rate (-12.7%) is 8.4 percentage points higher, with large-scale resettlement projects confirmed as the core driving factor. International cases further highlight differences in development paths: based on the elevation gradient management experience of the Tennessee River Basin in the United States, this study proposes designating the 175–185 m elevation zone as the core area for intensive industrial development in the reservoir area; however, comparative data from the Itaipu Reservoir Area in Brazil show that the development intensity of tourism land in the Three Gorges Reservoir Area (5.1%) is only 27.9% of that in Itaipu (18.3%), indicating significant room for improvement in the ecological tourism industry.”*

**7.Comment:**

Some paragraphs (such as section 3.1 and Section 3.6) are lengthy in language. It is recommended to simplify the sentence structure and highlight the key content.

**Response:**

We are grateful for your insightful feedback regarding our presentation of novel contributions in line 73. Your observation that "spatiotemporal" alone does not constitute methodological novelty has prompted us to re-examine and refine our discussion of research innovations. We fully agree that true novel contributions should be framed through original theoretical perspectives, methodological frameworks, or indicator systems. Accordingly, we have revised this section..The specific modifications are outlined below:

*“The data of the previous 30 years presented in Table 3 and Fig 3 indicate that the overall land use structure of the TGRR has been stable, with forest land and arable land being the dominant land use types. The distribution of arable land in the middle and upper reaches of the TGRR was relatively concentrated; moreover, the forest land was concentrated in the first section of the TGRR and the ranges of Mingyue, Zhongliang, Jinyun and Tongluo. Grasslands, as the third terrestrial land use type showed a sporadic distribution pattern in various areas of the TGRR, while construction land showed a relatively concentrated distribution, such as in the core urban region of Chongqing. In terms of quantitative changes, the land use types that showed an increasing trend during the research period were building land, water areas, and unused land, whereas cultivated land, forest land, and grassland showed a decreasing trend. Building land had the largest land category in the TGRR, increasing from 0.41% to 2.84%, with a net increase of 1837.28 km2. This increase was because of the rapid growth of the social economy in the TGRR, which has a large amount of construction land. Cultivated land was the largest land type in the TGRR, which decreased from 38.62% to 37.22%, with a net decrease of 961.45 km2. ~~Further, the land category with the smallest change was unused land. Owing to its small base and proportion of land area, the change was not significant; however, it showed a marginal increase, showing initially a trend of grouping and then declining, followed by an increasing and then a decreasing trend. This can be attributed to the continuous economic growth in the TGRR and continuous expansion of urban and rural planning scope, resulting in high demand for building land area and a large amount of development land.~~ ”*

*“The results of the analysis diagram of the social carrying system (Fig 6) indicated that in the social carrying ability system of land resources, with the rapid development of Chongqing’s direct administration and economy, the per capita area of public service facilities and urbanization rate showed a rapid increasing trend. In terms of population employment rate, there was an upward trend from 1986 to 1995, and a decrease from 1995 to 2007, partly due to the expansion of Chongqing's jurisdiction and the increase in population. An upward trend was also observed from 2007 to 2020. The natural population growth rate decreased from 1986 to 1995, increased from 1995 to 2007, decreased from 2007 to 2010, and slowly increased from 2010 to 2020.*

*Based on the trend results of evaluation values of land resource carrying ability of each subsystem in the TGRR (Fig 8), in the past 30 years, the social, economic, population, and ecological carrying abilities showed varying rates of increase. The ecological carrying ability had the largest fluctuation compared to the economic carrying capacity, mainly because of the unprecedented rapid economic growth of Chongqing since its direct administration in 1997. With economic growth, more resources have been invested in ecological environmental protection and governance, and progress has been made in the construction of an ecological civilization. The social and population carrying capacities of the research area showed an upward trend; however, the rate was not significant. On analyzing each period, we found that the overall situation of the four individual carrying capacities in the TGRR before 2000 was as follows: population carrying capacity was superior to social carrying capacity, and ecological carrying capacity was superior to economic carrying ability. ~~During 2000–2010, the overall situation of the four individual carrying capacities in the research area was: ecological carrying ability was superior to social carrying ability and economic carrying ability was superior to population carrying ability. In 2010, the overall situation of the four individual carrying capacities was: ecological carrying capacity was superior to economic carrying ability, and social carrying ability was superior to population carrying ability.~~”*

**8.Comment:**

Some formulas (such as Formula 1 and Formula 2) are too tightly packed. It is recommended to adjust them to be displayed in the center, and add a detailed explanation of the variables after the formulas.

**Response:**

Thank you to the reviewers for their valuable suggestions on the presentation of formulas! We have comprehensively optimized the formulas in the article.

**9.Comment:**

In section 2.2, The author mentions the accuracy of remote sensing image classification (94.52%) and Kappa coefficient, but does not explain how to conduct error analysis. It is suggested to supplement the error sources and verification details of classification accuracy.

**Response:**

We sincerely appreciate the valuable comments provided by the reviewers. We fully agree that a detailed error analysis and verification of classification accuracy are crucial for assessing the reliability of the results. The study area, the Three Gorges Reservoir Region, is a typical mountainous terrain characterized by steep slopes, deep valleys, and frequent cloud and fog conditions throughout the year. These factors constitute the core objective environmental background influencing the accuracy of remote sensing image interpretation. The primary source of error stems from image quality issues: frequent cloud cover and fog result in partial cloud coverage, mountain shadows (especially in deep valley areas), and interference from thin fog or haze in some temporal images, leading to pixel value deviations and blurred spectral information of ground features. Despite the use of multi-temporal image fusion and cloud mask techniques for preprocessing, residual cloud shadows or haze effects in some areas remain difficult to completely eliminate, inevitably introducing misclassification errors (e.g., misclassifying shadows as water bodies, or vegetation under haze as bare soil/built-up areas) or omission errors (e.g., inability to identify objects under clouds). Additionally, the 30m spatial resolution Landsat imagery used in this study faces significant mixed pixel issues when applied to the complex and fragmented land cover of the reservoir area, resulting in an increase in small patches, blurred boundaries, and category confusion. Topographic effects further exacerbate these issues: the steep terrain not only amplifies shadow ranges but also causes spectral confusion or misclassification due to differences in solar radiation reception between sunny and shaded slopes. This terrain-driven spectral variability interacts with the inherent spectral confusion of land cover: certain land cover types in the study area exhibit similar spectral characteristics in specific bands or seasons, such as sparse vegetation/shrubland and bare soil/built-up areas during the dry season, water bodies in different states (depth, turbidity), and newly afforested areas/young forests and grasslands or certain crops during specific growth stages. Finally, the representativeness of training and validation samples is a critical factor: in areas with steep terrain, poor transportation, or persistent cloud cover, obtaining sufficient quantities of high-quality field or high-resolution reference samples poses significant challenges, potentially leading to insufficient sample representativeness in these regions and thereby affecting local classification accuracy. In summary, the unique geographical and climatic conditions of the Three Gorges Reservoir area (mountainous terrain, frequent clouds and fog), limitations in image spatial resolution, spectral similarity of landforms and terrain-induced spectral variability, limitations of classification algorithms, and difficulties in sample acquisition collectively constitute the primary sources of error in the classification results of this study.*”*

**10.Comment:**

In section 3.5, The weight distribution is briefly described in table 8. It is suggested to supplement the reasonable discussion of the weight distribution and explain its influence on the comprehensive evaluation results.

**Response:**

We sincerely thank the reviewers for their valuable comments. We fully agree that clarifying the rationality of weight allocation and its impact on the results is crucial. The article has clearly stated in Section 2.3.3 (Land Resource Carrying Capacity Assessment Index System) that this study uses the Mean Squared Error Method (MSE Method, Wang Mingtao, 1999) from the objective weighting method to determine the weights of each indicator. The following key content has been added in Section 3.5 (near Table 8) to enhance the clarity of the discussion:. The specific modifications are outlined below:

*“Table 8 Weight values of evaluation indicators for land resource carrying ability in the TGRR based on the mean square error method.*

| **Indicator** | **Weight** | **Indicator** | **Weight** | **Indicator** | **Weight** | **Indicator** | **Weight** |
| --- | --- | --- | --- | --- | --- | --- | --- |
| X_1_ | 0.0673 | X_5_ | 0.0637 | X_9_ | 0.0635 | X_13_ | 0.0731 |
| X_2_ | 0.0586 | X_6_ | 0.0630 | X_10_ | 0.0532 | X_14_ | 0.0665 |
| X_3_ | 0.0612 | X_7_ | 0.0585 | X_11_ | 0.0604 | X_15_ | 0.0633 |
| X_4_ | 0.0565 | X_8_ | 0.0645 | X_12_ | 0.0654 | X_16_ | 0.0611 |

*This study determines weights using the mean square deviation method within the objective weighting method (Wang Mingtao, 1999), whose core principle lies in reflecting the information content and variability of the data itself: each indicator is treated as a random variable, with its value being the standardized value of the evaluation unit. The spatial variability of each indicator across all units is quantified by calculating the mean square deviation of its values across all units; the greater the variability (the larger the mean square deviation), indicates that the indicator has a stronger ability to distinguish the carrying capacity of different regions and contains a larger amount of information. After normalization, it is assigned a higher weight, ensuring that the weight allocation is derived from the intrinsic objective attributes of the data and avoids subjective interference. This weighting method based on spatial variability directly determines the sensitivity of the evaluation results to key differential indicators: high-weight indicators (i.e., indicators with significant spatial variability) have a decisive influence on the spatial distribution pattern of the final comprehensive evaluation value and carrying capacity grade. In this context, indicators with high spatial variability often correspond to core limiting factors or key advantageous factors affecting the reservoir area's carrying capacity. The application of the standard deviation method to assign higher weights to such indicators enables evaluation results to more accurately identify and focus on these key drivers of regional differences, thereby objectively revealing the primary causes of differentiation. Additionally, this objective method, which calculates weights based on actual data, enhances the reproducibility and stability of evaluation results.”*

*In this study, Landsat Thematic Mapper (LTM) false-color remote sensing image data (https://search.earthdata.nasa.gov) with an accuracy of 30m*30m were used. administrative division data were obtained from the National Geographic Information Center of China (http://www.ngcc. cn/ngcc/). In addition, population, economic, social, and ecological data were converted from Chongqing Statistical Yearbook and Hubei Provincial Statistical Yearbook (1986-2020) and related data such as land use. Land use types were categorized by supervised classification method, and the accuracy comparison results based on 688 randomly selected ground truth sample points showed that the accuracy was 94.52%. Comparison of the accuracy based on 688 randomly selected ground-truth sample points shows that the accuracy is 94.52% and the Kappa coefficients are also highly consistent, thus confirming the accuracy and scientific value of the data.”*

**11.Comment:**

In section 4, the article mentions that "ecological carrying capacity has been significantly improved", but does not specify which policies or projects (such as the project of returning farmland to forest) have had a direct impact on ecological carrying capacity. It is suggested to supplement relevant case analysis.

**Response:**

We sincerely appreciate the reviewers' attention to the driving factors of ecological carrying capacity in the Three Gorges Reservoir Area and their valuable suggestions. After carefully reviewing the original text multiple times, we found that the current version of the article does not directly use the phrase “ecological carrying capacity has been significantly enhanced” in Section 4 (Results and Discussion). The specific phrase pointed out by the reviewers may stem from their interpretation of the article's core conclusions or related trend descriptions. Nevertheless, we fully agree with the core value of the reviewers' opinions: thoroughly exploring the key policies and projects influencing the ecological carrying capacity of the Three Gorges Reservoir Area is crucial for understanding the driving mechanisms of regional ecological and environmental changes, enhancing the persuasiveness of research conclusions, and providing references for future management. This is indeed an area that can be further deepened in our discussion.. The specific modifications are outlined below:

“*(5)The primary reasons for the significant improvement in the ecological carrying capacity of the study area are as follows: The Grain-for-Green Program has played a foundational role in the reservoir area by increasing forest and grassland coverage, effectively controlling soil erosion, and enhancing water conservation capacity; the Natural Forest Resources Conservation Program is dedicated to protecting the integrity of existing forest ecosystems and maintaining biodiversity, holding significant long-term significance; the Three Gorges Reservoir Area Ecological Barrier Zone Construction and Reservoir Shore Zone Comprehensive Improvement Program focuses on stabilizing reservoir shores, managing the water level fluctuation zone, reducing sediment inflow into the reservoir, and ensuring water quality safety, achieving notable targeted results; and small watershed comprehensive management has played a positive role in controlling soil erosion at its source and improving local ecological environments. The coordinated advancement of these ecological projects has provided strong support for the restoration and sustainable development of the Three Gorges reservoir area's ecological system.*”

**12.Comment:**

In section 5, The conclusion section should further refine the highlights of the research and avoid duplication with the discussion section. For example, the specific impact of land use change on carrying capacity and policy implications can be highlighted.

**Response:**

Thank you for your valuable comment. According to your suggestion, we have further refined the highlights of the research in Section 5 (Conclusion), with a focus on emphasizing the key findings and their broader significance without duplicating content from the Discussion section. Specifically, we have strengthened the discussion on the specific impact of land use change on carrying capacity and elaborated on the policy implications derived from our findings. These revisions aim to enhance the clarity and contribution of the conclusion, and we appreciate your guidance in improving the quality of our paper. The specific modifications are outlined below:

*“5.2 Recommendations*

*~~In response to the changes in land resources in the TGRR, it is recommended to strengthen land use planning and supervision, with a focus on preserving farmland and ecological land while rationally allocating land for construction. This approach aims to enhance the social, economic, and ecological carrying capacities of the region. Furthermore, promoting green development models, fostering technological innovation, and improving land resource utilization efficiency are crucial steps. Additionally, by refining policies and regulatory frameworks, we can ensure the sustainable utilization of land resources, thereby laying a solid foundation for the region's sustainable development.~~*

*In light of the ongoing changes in land resources within the Three Gorges Reservoir Region (TGRR), this study underscores the critical need for strengthened land use planning and regulatory oversight. The transformation of farmland and ecological land into construction land has been identified as a key factor constraining the region’s social, economic, and ecological carrying capacities. Therefore, preserving high-quality arable land and essential ecological zones should be prioritized, while construction land allocation must be guided by principles of sustainability and spatial optimization.Moreover, the findings highlight that integrated land management strategies—such as promoting green development models, advancing technological innovation in land-use efficiency, and implementing adaptive governance—are instrumental in enhancing regional resilience and long-term carrying capacity. To institutionalize these practices, it is imperative to refine existing policies and regulatory frameworks, ensuring they align with the ecological sensitivity and developmental needs of the TGRR.This study thus provides actionable insights for decision-makers, offering a science-based foundation for sustainable land resource management and policy formulation in ecologically vulnerable regions facing rapid land use transitions.”*

# We deeply appreciate the time and effort these reviewers have dedicated to evaluating our manuscript, and we eagerly await any further feedback or suggestions.
