# Supplementary material for: Spatiotemporal characteristics and optimization strategies of land use and land resource carrying capacity in the three gorges reservoir region (1986–2020)
Source: PLoS One. 2025 Aug 21;20(8):e0330461. doi: 10.1371/journal.pone.0330461 (PMC12370055; doi:10.1371/journal.pone.0330461)
Supplement: Attachment — Submitted filename: Response to Reviewers-R4.docx [file pone.0330461.s005.docx]

Point to Point Responses

**Paper Ref. No.:PONE-D-24-37148R3**

**Title:**Spatiotemporal Characteristics and Optimization Strategies of Land Use and Land Resource Carrying Capacity in the Three Gorges Reservoir Region(1986–2020).

Thanks so much for all the kind help to this manuscript. We have analyzed the valuable comments from the editor and reviewers#3 carefully, and tried our best to revise the manuscript. We appreciate the thoughtful review and constructive feedback provided by two viewers.We agree with the reviewer's suggestions and have incorporated the recommended changes into the manuscript.These comments have improved the quality of the paper immensely. The point to point responses are listed as follows.

# Comments（Editor and Reviewer#3）:

Thanks very much for your comments. According to the following valuable comments and suggestions, we have tried our best to revise the manuscript, and these comments have substantially improved the quality of the paper.

**1.Comment:**

In keywords, the spatiotemporal characteristics and evaluation could be integrated together, such as evaluation of spatiotemporal characteristics.

**Response:**

We appreciate the reviewer’s suggestion. Accordingly, we have revised the keywords by integrating “spatiotemporal characteristics” and “evaluation” into a single term: “Evaluation of spatiotemporal characteristics”. This change enhances clarity and better reflects the focus of our study.

*“***Keywords:** Land use; Land Resource Carrying Capacity; Evaluation of spatiotemporal characteristics; Three Gorges Reservoir Region *”*

**2.Comment:**

In introduction, the authors shown three major frontier advances. However, these advances are great, your innovation is not enough compared these advances.

**Response:**

Thank you for your valuable feedback on our research. We agree with your point that, although we mentioned three cutting-edge developments in the introduction, we need to further emphasize our innovation to highlight our unique contributions compared to these developments. Based on your suggestion, we have added the following content to the introduction to more clearly demonstrate the innovative points and unique value of this study.The specific revisions are as follows:

*“In recent years, land use change and its impact on ecosystems and socio-economics have become a global research hotspot. Particularly in the Three Gorges Reservoir Region (TGRR), land use changes have been particularly pronounced due to large-scale infrastructure development and socioeconomic progress. While existing studies have examined land use change trends using multi-source data and long-term time series analysis, assessed land resource carrying capacity through integrated evaluation models, and proposed optimization strategies for sustainable development, these studies often focus on short timeframes or specific types of land changes, lacking systematic integration of multiple influencing factors and concrete, actionable strategies. The innovation of this study lies in: we utilized Landsat imagery and statistical yearbook data from 1986 to 2020 to systematically analyze the spatiotemporal evolution characteristics of land use changes in the Three Gorges Reservoir Region and generated high-precision land use maps; Based on the Analytic Hierarchy Process (AHP) and the mean square deviation decision-making method, we constructed a comprehensive land resource carrying capacity evaluation model that considers social, economic, demographic, and ecological factors; and through an in-depth analysis of the mechanisms by which different driving factors influence land use changes, we proposed targeted optimization strategies aimed at promoting the efficient use of land resources and ecological protection in the Three Gorges Reservoir Area, thereby advancing regional sustainable development.”*

**3.Comment:**

The sub-sections are too much. It should be integrated. Such as 3.2,3.3 and 3.4.;3.6 and 3.7. 3.5.1, 3.5.2,3.5.3.

**Response:**

We agree with the reviewer’s suggestion regarding the excessive number of subsections. To improve readability and logical flow, we have merged several sections as follows:

Sections 3.2, 3.3, and 3.4 were combined into a new section titled “Land Use Dynamics and Accuracy Assessment in the TGRR”;

Subsections 3.5.1–3.5.3 were integrated into “Verification of land use classification results”;

Sections 3.6 and 3.7 were merged into “Integrated Assessment of Land Resource Carrying Capacity in the TGRR”.

These changes enhance the structural coherence of the methodology section.

**4.Comment:**

3.8 should be moved to discussion.

**Response:**

Thank you for this constructive suggestion. We have relocated the content originally presented in Section 3.8 to the Discussion section, where it now serves to interpret the underlying mechanisms and contextualize our findings within broader regional and policy-relevant perspectives.

**5.Comment:**

The relevant references should be citied as follow

Spatial and temporal heterogeneity of urban land area and PM2.5 concentration in China. Urban Climate,2022,45:101268. doi:https://doi.org/10.1016/j.uclim.2022.101268.

Challenges and considerations of applying nature-based solutions for furture mega-cities: Implications for Karachi as a Sponge City.Human Settlements and Sustainability,2025,1:50-61. doi:https://doi.org/10.1016/j.hssust.2025.02.002

**Response:**

We thank the reviewer for recommending these relevant references. Both papers have been added to the reference list and appropriately cited in the Discussion section, particularly when discussing spatial heterogeneity and sustainable urban development strategies.

# We deeply appreciate the time and effort these reviewers have dedicated to evaluating our manuscript, and we eagerly await any further feedback or suggestions.
